# Genome-wide identification of short-chain dehydrogenases/reductases genes and functional characterization of *ApSDR53C2* in melanin biosynthesis in *Arthrinium phaeospermum*

**DOI:** 10.3389/fmicb.2025.1532162

**Published:** 2025-01-30

**Authors:** Jiao Liao, Yisi Wang, Han Liu, Sijia Liu, Peng Yan, Hang Chen, Shujiang Li

**Affiliations:** ^1^College of Forestry, Sichuan Agricultural University, Chengdu, China; ^2^Ganzi Institute of Forestry Research, Kangding, China; ^3^National Forestry and Grassland Administration Key Laboratory of Forest Resources Conservation and Ecological Safety on the Upper Reaches of the Yangtze River & Forestry Ecological Engineering in the Upper Reaches of the Yangtze River Key Laboratory of Sichuan Province, Chengdu, China

**Keywords:** *Arthrinium phaeospermum*, Bambusa pervariabilis × Dendrocalamopsis grandis, SDR, melanin, gene function

## Abstract

**Introduction:**

*Arthrinium phaeospermum* can cause large areas wilted and death of *Bambusa pervariabilis* × *Dendrocalamopsis grandis*, resulting in serious ecological and economic losses. Previous studies found that the appressorium of *A. phaeospermum* must form to invade the host cells and cause disease. A short-chain dehydrogenase/reductase gene has been shown to maintain the osmotic pressure of the appressorium by synthesizing fungal melanin to penetrate the plant epidermis and cause disease. The SDR gene family of *A. phaeospermum* was found to be highly expressed during the penetration in the transcriptome sequencing results. Still, the relationship with melanin biosynthesis of *A. phaeospermum* is not clear.

**Methods:**

We aimed to predict the biological function of the SDR gene family in *A. phaeospermum*, identify key *ApSDR* genes with pathogenic roles, and explore the pathogenic mechanism. We have characterized the SDR family of *A. pheospermum* bioinformatically. Candidate *ApSDRs* screened by transcriptome sequencing were compared by qPCR experiments to obtain key *ApSDRs* that may play an important role in infestation and adversity resistance. Knockout mutants, the co-knockout mutant, and backfill mutants of key *ApSDRs* were obtained for phenotypic and stress conditions analysis. We explored and validated the pathogenic mechanisms through cellulose membrane penetration experiments and analysis of melanin-related gene synthesis levels.

**Results and discussion:**

180 *ApSDRs* were identified bioinformatically. After screening six candidate *ApSDRs* with noticeably elevated expression using transcriptome sequencing, qPCR experiments revealed that ApSDR53C2 and ApSDR548U2 had the highest expression. The results of phenotypic and stress conditions analysis indicate that ApSDRs are critical for the growth, development, stress response, and fungicide resistance of *A. phaeospermum*. The pathogenicity analysis revealed that *ApSDR53C2* and *ApSDR548U2* play important roles in virulence, with *ApSDR53C2* having a stronger effect. A comparison of melanin synthesis levels between wild-type and Δ*ApSDR53C2* strains showed that *ApSDR53C2* positively regulates melanin biosynthesis to promote penetration. The findings demonstrate that ApSDRs are essential for *A. phaeospermum* to withstand stress and facilitate melanin biosynthesis, which in turn contributes to its virulence.

## Introduction

A significant new bamboo species used for ecological building in China’s Yangtze River Basin is *Bambusa pervariabilis* × *Dendrocalamopsis grandis*, which has high economic, ecological, and social benefits (Fang et al., 2022). Nevertheless, a sizable tract of hybrid bamboo dieback was discovered in Sichuan Province’s introduction region in 2003, converting cropland back to woods. Li et al. (2013) identified the disease as hybrid bamboo blight and determined the pathogen to be *Arthrinium phaeospermum*. Currently, studies on hybrid bamboo blight mainly focus on the disease pattern, pathogen biology, and pathogen protein toxin virulence. Other areas of research include the identification of biocontrol bacteria and the genomics of transcriptome, proteome, and metabolome of hybrid bamboos after pathogen infestation and toxin induction (Vijayakumar et al., 1996; van Eijk, 2005; Khan et al., 2009; Shrestha et al., 2011; Li and Zhu, 2014; Liao et al., 2022). On the other hand, *A. phaeospermum*, as a facultative necrotrophic pathogen, mainly lives by parasitizing hybrid bamboo, but after the death of the tissue cells at the invasion site, it will live in saprophytic life with the help of dead hybrid bamboo tissues. Its infestation mainly uses conidia to attach to the surface of plant tissues. The conidia, after sprouting bud tubes, specialize mycelium from the bottom to invade the host cells by forming appressorium at the tip after maturation (Li et al., 2013). Studies on the pathogenicity genes of this pathogen have only dealt with the effector genes acting in the symptom appearance phase (Fang et al., 2021, 2022, 2023; Yan et al., 2022), and the key genes of its penetration phase remain unclear.

The term short-chain dehydrogenases/reductases (SDRs) was first coined in 1991 (Persson et al., 1991), and approximately 25% of dehydrogenases belong to the SDR superfamily (Kallberg and Persson, 2006). The SDRs consist of NAD (P) (H)-dependent oxidoreductases and their sequences are usually around 250 amino acid residues. Their active site consists of a catalytic triad or tetrad composed of tyrosine (Tyr), lysine (Lys), serine (Ser), and asparagine (Asn) (Ghosh et al., 1994; Filling et al., 2002). Although the sequences of the SDR superfamily members are highly heterogeneous, they all contain a conserved “Rossmann-fold” structure (Kavanagh et al., 2008). The SDR superfamily is widespread in eukaryotes (Jörnvall et al., 1999) and is of multifunctional and fundamental importance in metabolic processes (Persson et al., 2009). The enzymes of this gene family typically catalyze NAD (P) (H)-dependent reactions with diverse substrates (Oppermann et al., 2003; Kallberg et al., 2010). Short-chain dehydrogenases have diverse catalytic functions and can regulate a wide range of physiological and biochemical reactions in organisms by participating in substance metabolism and redox reactions (Persson et al., 2003; Roth et al., 2018). Members of the SDRs have been shown to influence plant growth, development, and response to adversity by participating in primary and secondary metabolism in plants (Stavrinides et al., 2018). In addition, SDRs play a variety of important roles in cell differentiation and signal transduction (Kallberg et al., 2002). Studies on the function of the SDRs in plant pathogen pathogenesis are comparatively scarce. A member of the SDRs, 3HNR, is a key gene in the pathogenesis of *Magnaporthe oryzae* (Kwon et al., 2010); another SDR gene contributes to *Rhizopus erythropolis*’s biosynthesis of fumonisins (Butchko et al., 2003). The results of transcriptome sequencing showed that the expression of *ApSDRs* significantly increased during the penetration phase of *A. phaeospermum* (Fang et al., 2021). It is hypothesized that *ApSDRs* play a role in the contact and invasion phases during the process of infesting hybrid bamboo as a pathogenicity factor.

Melanin plays an important role in both adversity resistance and infestation by fungi, many of which are unable to produce mechanical forces sufficient to penetrate the host due to the inability to form melanized appressorium, which in turn affects pathogenicity (Luo et al., 2012). Fungi synthesize melanin mainly through the 1, 8- dihydroxynaphthalene (DHN) pathway and the 3, 4- dihydroxyphenylalanine (DOPA) pathway (Polak, 1990). Melanin in *A. phaeospermum* is synthesized via the DHN pathway which begins with the precursor molecule acetyl coenzyme a or malonyl coenzyme and is catalyzed by a polyketide synthase (Pks) to form tetrahydroxynaphthalene (THN), followed by a series of reductions and polymerizations to melanin (Bell and Wheeler, 1986; Chen et al., 2015). The genes widely recognized to be involved in melanin biosynthesis in the DHN pathway are CMR, Lac, THN-reductase, PKS, and T4HN-reductase (Tsai et al., 1999; Langfelder et al., 2003; Schätzle et al., 2012; Jia et al., 2021). The SDR genes and melanin biosynthesis are closely related. The fungal pathway for the synthesis of 1,8-DHN consists of type I PKS, the SDR superfamily of naphthol reductase (HNR) and sickle ketone dehydrogenase (SD); a member of the SDR gene family, 1,3,8-trihydroxynaphthalene reductase (3HNR), is an essential enzyme involved in the biosynthesis of fungal melanin (Sone et al., 2018).

Thus, using the whole genome sequence, a variety of bioinformatics analysis tools, and Real-time PCR, the SDR family of *A. phaeospermum* was identified and the *ApSDRs* that might have a pathogenicity role were subsequently screened out. The functions of the candidate pathogenicity *ApSDRs* were confirmed through protoplast-mediated gene knockout and backfill techniques. The specific pathogenicity mechanism of the more virulent gene *ApSDR53C2* was further explored by cellulose membrane penetration assay and melanin synthesis level analysis. For the first time, a family of SDRs in fungi has been methodically discovered through bioinformatics analysis, and this serves as a guide for investigating the pathogenicity function of SDRs in fungal pathogens.

## Materials and methods

### Identification of ApSDR genes

The genome sequence of *A. phaeospermum* was downloaded from NCBI resources (Li et al., 2019) (NCBI GenBank accession number QYRS00000000). The SDR protein sequences of *Saccharomyces cerevisiae*, *Fusarium oxysporum*, *Magnaporthe oryzae*, and *Rosellinia necatrix* were downloaded from NCBI^1^ and were used for BLASTP search and phylogenetic tree construction. Using BLASTP, the 23 amino acid sequence of the *ScSDRs* was used as a query sequence to identify homologous genes (e < 1 × 10^–5^). The genome set of predicted proteins was selected by HMMER3^2^ using three Pfam HMMs: PF00106, PF01370, and PF01073. The NCBI CDD^3^ and SMART^4^ were used to analyze ApSDRs protein sequences in order to predict protein structures, remove redundant sequences, hand-select candidate SDR-encoding genes and perform functional annotation.

### Multiple sequence alignment and phylogenetic analysis

The protein sequences of 180 ApSDRs, 23 ScSDRs, 21 FoSDRs, 6 MoSDRs, and 36 RnSDRs were analyzed in a multi-sequence comparison by ClustalW algorithm (Larkin et al., 2007). Using MEGA 11.0^5^, a phylogenetic tree with 1,000 bootstrap repetitions was constructed using the neighbor-joining method.

Based on the structural characteristics of the sequences, the SDR superfamily is usually classified into seven categories: Classical, Extended, Intermediate, Complex, Divergent, Unassigned, and Atypical (Labesse et al., 1994; Persson and Kallberg, 2013). Nowadays, SDRs segmentation and nomenclature are frequently based on Hidden Markov Models (HMMs), with each family having a name SDR followed by a number and a letter to indicate the type (Persson et al., 2009). All *ApSDRs* were categorized and named according to the HMMs and phylogenetic tree.

### Motif and gene structure analysis

Conserved motifs were analyzed using MEME^6^ (Bailey et al., 2009). The maximum number of motifs was set to 10, and the length of motifs was set to 10∼50 amino acids. NCBI CDD was used to predict the conserved structural domains of proteins (see text footnote 3). The exon-intron organization was predicted by comparing the coding sequence with the corresponding DNA sequence using Gene Structure Display Server 2.0 (GSDS 2.0).^7^ The results were expressed using TBtools software (Chen et al., 2020).

### Chromosomal distribution and synteny correlation analysis

The chromosomal location of *ApSDRs* was analyzed using genome annotation files. MG2C was used to map the *ApSDRs*’ precise location on the chromosome.^8^

Homologous gene pairs of the SDR gene family in *A. phaeospermum*, *F. oxysporum*, *Apiospora arundinis*, and *Arthrinium hydei* were identified using OrthoMCL software,^9^ with parameters set to allow a minimum protein length of 10 and a maximum percentage of stop codons of 20%. Utilizing the default parameters, gene pair covariance was determined using MCScanX software.^10^

Based on the whole genome sequencing and annotation results of *A. phaeospermum*, the genes of *ApSDRs* were used as foreground genes, and all the genes of *A. phaeospermum* were used as background genes by using the OmicShare cloud platform.^11^ GO and KEGG analysis and mapping of SDR gene family in *A. phaeospermum*.

The protein interaction network database STRING^12^ was used to predict protein interactions within gene families for all SDRs in *A. phaeospermum* to obtain protein interaction networks. The results were exported to Cytoscape software for further annotation and mapping.

Signal peptide was predicted using SignalP-6.0.^13^ Transmembrane structure was predicted using TMHMM-2.0.^14^ Protein hydropathicity/hydrophobicity was mapped using ProtScale.^15^ Protein tertiary structure was predicted using SWISS-MODEL.^16^

### Sample processing for screening of candidate SDR genes and expression pattern analysis

Transcriptome sequencing data of *A. phaeospermum* infesting *B. pervariabilis* × *D. grandis* at 0, 7, 14, and 21 days were used to collect the expression data for all *ApSDRs* (Fang et al., 2022) (the original data were published in the NCBI Sequence Read Archive (SRA) under the accession number SAMN19312317). After selecting the top 30 *ApSDRs* with the highest expression, we analyzed their data at different times of pathogen infestation in comparison with the control (0 days). Then, we used Heml software after taking the log2 value, analyzed the differences in the expression levels of the SDR family genes during the infestation of *A. phaeospermum*, and screened out the possible key pathogenicity factors that involved in the invasion of the hybrid bamboos.

A PDA medium coated with 200 μL of sterile hybrid bamboo tissue fluid (T1) and 200 μL of sterile deionized water (CK) was used to inoculate *A. phaeospermum*. The mycelium was harvested after the strains were incubated for 7, 14, and 21 days at 25°C (Fang et al., 2021). Three duplicates of each sample were made.

*A. phaeospermum* was treated with 2 mg/mL Congo red, 2 mol/L sodium chloride (NaCl) and 40 mmol/mL hydrogen peroxide (H_2_O_2_), respectively, and an equal volume of water was used as the control. The samples were taken at 25°C for 10 days. Each sample was replicated 3 times.

Using the Tubulin of *A. phaeospermum* as the internal reference gene, primers for six candidate genes and Tubulin gene were designed using Primer Premier 5.0 software (Supplementary Table S1). The 2^–ΔΔCt^ method was used to evaluate the data (Livak and Schmittgen, 2001).

### Construction of ApSDR gene deletion mutants

When the accumulation of geneticin is 100 μg/mL or hygromycin reaches 350 μg/mL, *A. phaeospermu*’s mycelium stops growing completely (Fang et al., 2021). Therefore, hph and KanMx genes were selected as screening markers for knockout and backfill. The strategy employed in this study for the knockout of the SDR gene is shown in Supplementary Figure S1. The homology arms of *ApSDR53C2* and *ApSDR548U2* were designed with a length of 1,500 bp. For knockout backfill, the *ApSDR53C2* and *ApSDR548U2* genes were linked to the kanMx gene by complementary base pairing and fusion PCR. Then, the fusion fragments were ligated to the upstream and downstream homology arms of the target genes. Primers were displayed in Supplementary Table S2.

### Preparation of protoplasts

Enzymatic digestion: *A. phaeospermum* was inoculated on PDA medium and incubated at 25°C for 3 days. 5 agar blocks were scratched from the edge of the colony inoculated in PDB at 180 rpm and incubated at 25°C for 3 days. Then filtered through a single layer of mirocloth to collect the hyphae, and rinsed with 1.2 M KCl 5 times to obtain clean hyphae for spare. Another 0.1 g of lysing enzymes and 0.5 g of driselease enzymes were in a small beaker, added 20 mL of external 1.2 M KCl, added magnetic beads stirred for 10–15 min, transferred to a 50 mL centrifuge tube, rotated horizontally at 4°C, 3,500 rpm, up and down acceleration of 9, centrifuged for 10 min. Pouring back the supernatant into a small beaker, and filtered through a bacterial filter. The enzyme solution was sterilized and set aside. Transfer the mycelium into the enzyme solution, seal it, and put it into the shaker at 30°C, 70 rpm for 8 h. Microscopic examination: take out the cultured protoplasts, aspirate a drop on the slide, and examine it microscopically. A double layer of mirocloth was used to filter it. 25 mL of 1.2 M KCl stabilized osmotic solution rinsed to collect the filtrate, and centrifuged for 10 min at 4°C, 3,000 rpm, with an up and down acceleration of 5. Suction beating was used to mix the protoplasts, and the supernatant was disposed of. To clean the tube walls, 15 mL of KCl stabilizing osmotic solution was then added. After centrifuging the tubes once more for 10 min, 1 mL of STC buffer was added. Microscopic examination, according to the concentration of protoplasts will be divided into 2–5 centrifuge tube spares.

### Phenotypic and stress analysis of transformant strains

In order to investigate the differences in the sensitivity of the wild-type (WT) strain and the knockout and backfill transformant strains to cell wall stress, hyperosmotic stress, endoplasmic reticulum stress, and extracellular oxidative stress, the WT strain and the knockout and backfill transformant strains were inoculated with PDA medium containing 2 mg/mL of Congo red, 0.01% sodium dodecyl sulfate (SDS), 2 mol/L NaCl, 5 mmol/L dithiothreitol (DTT), and 40 mmol/L H_2_O_2_ on PDA medium.

To investigate the differences in the responses of the WT strain and the transformant strains to metal ions, different concentrations of MgSO_4_, MnSO_4_, and ZnSO_4_ were added to the PDA medium, and the concentrations of MgSO_4_ were set as follows: 0, 1, 2, 3, and 4 mM; the concentrations of MnSO_4_ were set as follows: 0, 5, 10, 15, and 20 mM; the concentrations of ZnSO_4_ were set to 0, 5, 10, 15, and 20 mM.

To compare the differences between the WT strains and the transformed strains in terms of resistance to different fungicides, dimethyl sulfoxide was used as a solvent to solubilize the agents and added into the PDA medium to adjust the medium to different concentrations for the determination of the resistance of the strains. Tricyclazole and phthalide were added to the medium to study the differences in the response of WT and transformed strains to osmotic-resistant fungicides, and the concentrations were set as follows: 0, 25, 50, 100, 200 μg/mL and 0, 12.5, 25, 50, 100 μg/mL. Azoxystrobin was added to the medium to study the differences in the response of WT and transformed strains to Qi inhibitors, and the concentrations of azoxystrobin were set as follows. 0, 12.5, 25, 50, 100 μg/mL.

The above experimental groups were incubated at 25°C for 5 days and then observed and photographed, and the colony diameters were measured and analyzed. To measure particular differences between pairs of means, all assays were conducted three times, and all data were analyzed using SPSS 16.0. Statistical significance was defined as a p-value of less than 0.05.

### Pathogenicity test

Forty uniformly growing 2-year-old *B. pervariabilis* × *D. grandis* plants were chosen. Five plants were chosen at random, and the WT strain’s mycelial suspension was sprayed on each plant’s upper shoots. The remainder were incubated with the mycelial suspension of mutants Δ*ApSDR53C2*, Δ*ApSDR548U2*, Δ*ApSDR53C2*: *ApSDR548U2*, Δ*ApSDR53C2*+, Δ*ApSDR548U2*+, Δ*ApSDR53C2*: *ApSDR548U2*+, and sterile water. The inoculated branches were bagged moisturized and sprayed every 12 h. After 25 days, the incidence was examined in order to determine the disease index.

The disease grading standard is shown in Supplementary Table S3. Disease index = (Σ (disease grade × number of diseased branches)/(total number of branches × highest disease grade) × 100.

### Function analysis of participation in melanin biosynthesis, cellulose membrane penetration

The WT strains and transformant strains were inoculated onto PDA mediums covered with sterile cellulose membrane, and were incubated at 25°C with alternating light and darkness for 4, 7, and 14 days, and then the cellulose membrane was removed and incubated for 4, 7, and 14 days, and the phenotypic changes were observed. Using a stereomicroscope (Nikon P-PS32), the colonies on the 14 days of cellulose membrane induced culture were observed, and the magnification of 2x and 5x objective lenses were selected. Photographs were taken to record the observations. After cellulose membrane induction for four, seven, and fourteen days, mycelium samples were prepared. RNA was extracted, and cDNA was obtained by reverse transcription. Primers of melanin synthesis-related gene and internal reference gene (Tubulin) for qPCR were displayed in Supplementary Table S4. cDNA was utilized as the template. The 2^–Δ^ ^Δ^ *^Ct^* method was used to evaluate the data.

PDA mediums with tricyclazole concentrations of 0, 12.5, 25, and 50 μg/mL were prepared, respectively, and inoculated with WT and transformed strains after covering with sterile cellulose membrane. Then inverted at 25°C for 7 days. The expression levels of melanin-related genes were analyzed and recorded under the different conditions of treatment.

## Results

### Identification of SDRs in *A. phaeospermum*

Using blast and HMMER3, 213 *ApSDR*s were found in *A. phaeospermum*. The structural domains of the genes that encoded these *ApSDR*s were then annotated, and the genes lacking the adh_short domain were eliminated, leaving 180 *ApSDR*s.

### Phylogenetic relationships and functional annotation of ApSDRs

**FIGURE 1:**
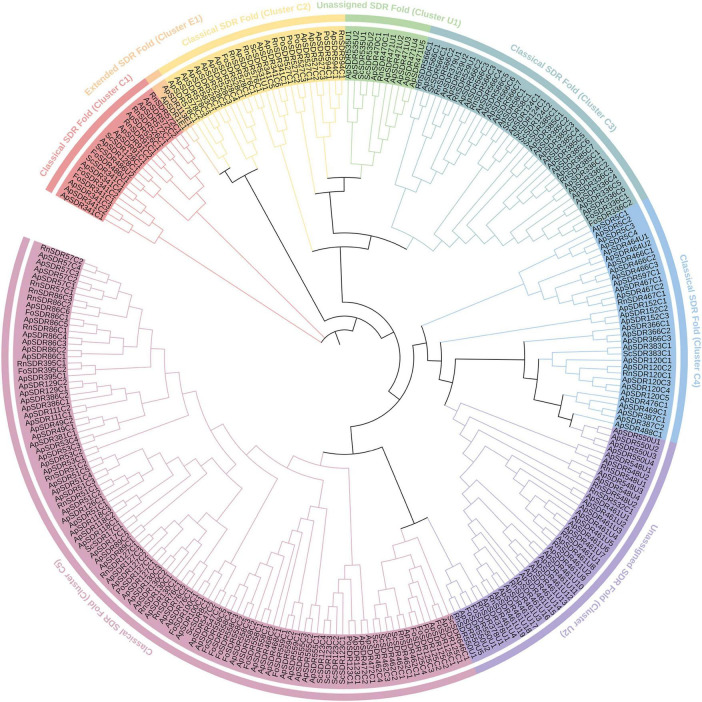
Phylogenetic tree of *ApSDR* gene family. Lines of various colors indicate groupings of corresponding colors and black lines indicate trunks within the tree.

According to the Hidden Markov Model and phylogenetic tree (Figure 1), all SDR genes in *A. phaeospermum* were classified, renamed, and categorized into three major groups (Classical, Extended, and Unassigned) and eight subgroups, among which the Classical type has the most *ApSDR*s with 141, the Extended type has 2, and Unassigned type has 37. In addition, *ApSDR*s in the classic type were further categorized into 5 subgroups (clusters C1, 2, 3, 4, and 5) containing 10, 15, 24, 29, and 63 members, respectively. Two subgroups (clusters U1 and U2) with 8 and 29 members were further divided from *ApSDRs* in the unassigned phenotype.

**FIGURE 2:**
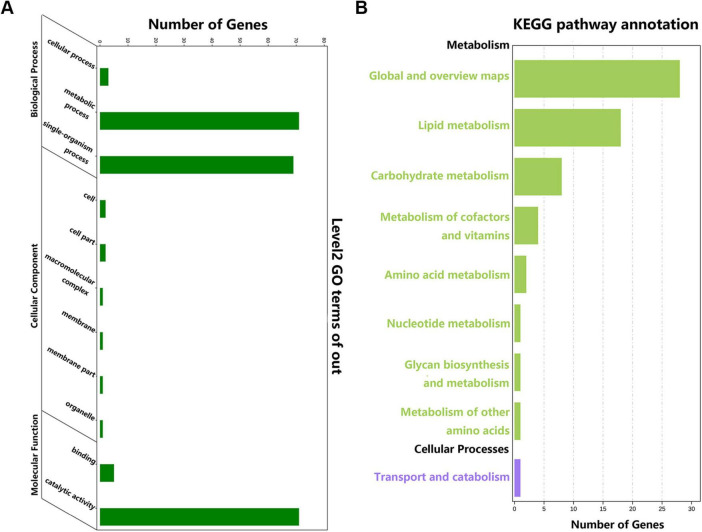
**(A)** GO analysis of *ApSDRs*. **(B)** KEGG analysis of *ApSDRs*.

We successfully annotated 180 *ApSDR*s into GO categories (Figure 2A). These *ApSDR*s were enriched in “metabolic process” (GO:0008152), “single-organism process” (GO:0044699), and “catalytic activity” (GO:0003824). As shown in Figure 2B, analysis of 180 *ApSDR*s KEGG-enriched pathways revealed that *ApSDR*s were mainly enriched in KEGG-enriched pathways such as global and overview maps, lipid metabolism, carbohydrate metabolism, and metabolism of cofactors and vitamins.

### Structural and motif localization analysis of ApSDRs

As shown in Supplementary Figure S2, ten conserved motifs ranging in size from 11 to 35 amino acid residues, numbered 1 through 10, were obtained and displayed in colored boxes.

Among the conserved motifs of ApSDR proteins, Motif 1, Motif 3, and Motif 4 encode conserved structural domains in the SDR gene family: the NAD (P)-binding Rossmann-fold. Motif 1 or Motif 3 can be observed in all ApSDRs, suggesting that all of them belong to the short-chain dehydrogenase/reductase (SDR) superfamily. Other conserved motifs (Motif 2, Motif 4–10) are also present in ApSDR proteins, ranging in number from 1 to 9.

Analysis of the conserved structural domains of *ApSDR* members showed that all members have NADB_Rossmann superfamily structural domains. Analysis of the exon-intron structures of 180 *ApSDRs* showed that the number of exons and introns in the same group was relatively conserved. The *ApSDRs* belonging to the classical SDR fold, on the other hand, had significantly more exons and introns than those belonging to the extended SDR fold, and the unassigned *ApSDRs*, suggesting that the functions of *ApSDRs* belonging to the classical SDR fold may be different from those of the other two groups.

It was discovered that, despite the low levels of amino acid sequence similarity, the *ApSDRs* were still highly conserved in the important amino acid sites. Nearly all of them contained the conserved motif TGxxxGxG at the N-terminal, which defines the cofactor binding site, and is a relatively conserved glycine-rich region (Oppermann et al., 2003). In this motif, amino acids 1, 2, 6, and 8 were also found to be highly conserved, as valine (T) and glycine (G), respectively (Supplementary Figure S3).

### Chromosomal distribution and synteny correlation analysis of ApSDRs

**FIGURE 3:**
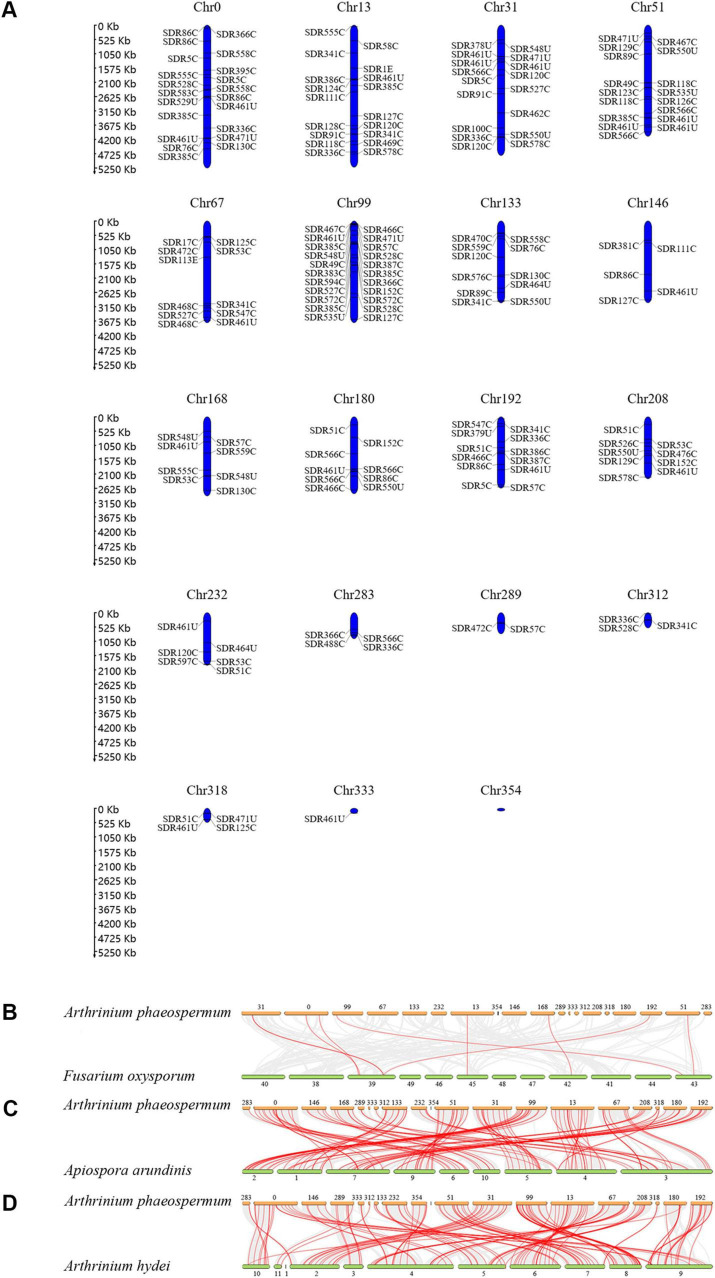
**(A)** Distribution of SDRs in the chromosomes of *A. phaeospermum*. **(B–D)** The orthologous relationship of SDRs between *A. phaeospermum* and *F. oxysporum*, *A. arundinis*, or *A. hydei*. Gray lines indicate gene pairs that are co-linear between the two species. Red lines indicate homologous SDR gene pairs between the two species.

These 180 *ApSDRs*, widely dispersed on 18 of the 19 *A. phaeospermum* chromosomes, ranged from 1 to 22 *ApSDR* genes per chromosome, as Figure 3A illustrates. With 22 *ApSDRs*, chromosome Chr99 has the greatest distribution of *ApSDRs*. There were no *ApSDR* found on Chr354.

The orthologous relationship of SDR genes between *A. phaeospermum* and *F. oxysporum*, *A. arundinis*, or *A. hydei* was examined (Figure 3B). The results revealed that there were 9 pairs of homologous genes identified between *A. phaeospermum* and *F. oxysporum*, 123 pairs between *A. phaeospermum* and *A. arundinis*, and 141 pairs between *A. phaeospermum* and *A. hydei*. There is low synteny and fewer homologous SDR in *F. oxysporum* species because they are in different genera and families, whereas there is high synteny in *A. phaeospermum* and *A. hydei* species because they belong to the same genus (Li et al., 2019). The degree to which homologous genes persist on corresponding chromosomes (synteny) and in conserved orders (collinearity) throughout evolution can be determined by comparing the genomes of related eukaryotic organisms. The more homologous gene pairs a gene has between species, the more important the gene is likely to be in the phylogeny of that gene family (Coghlan et al., 2005). Eight *ApSDRs* (*ApSDR566C1*, *ApSDR86C5*, *ApSDR124C1*, *ApSDR49C2*, *ApSDR76C1*, *ApSDR126C1*, *ApSDR51C1*, and *ApSDR53C4*) are related to at least three homologous gene pairs, indicating that the phylogeny of the SDR gene family may be significantly influenced by these genes.

### Prediction of ApSDR protein interactions

**FIGURE 4:**
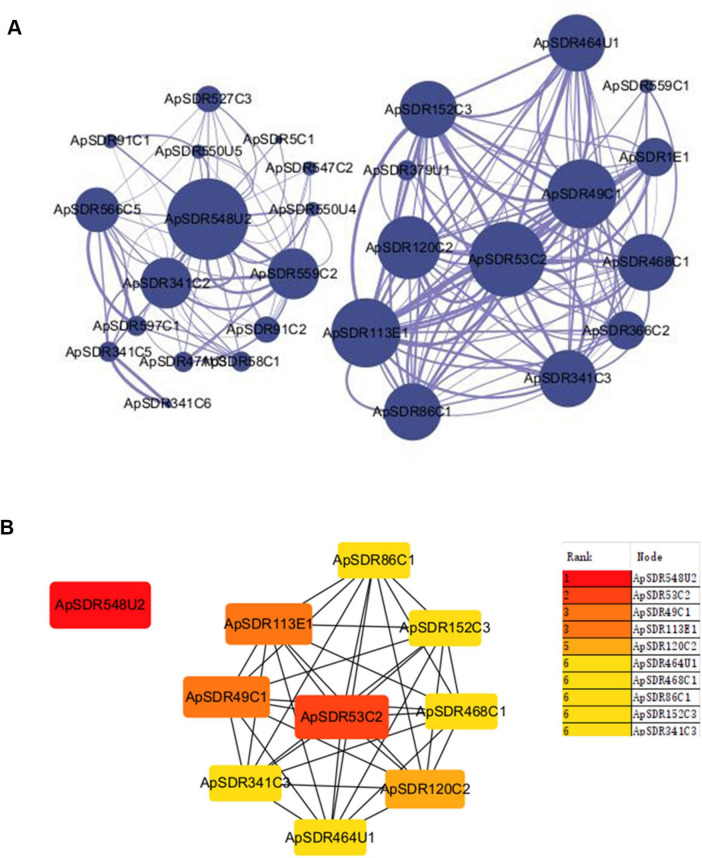
Protein interaction network analysis diagram of ApSDRs. **(A)** Visualization diagram of the PPI network of ApSDRs in Cytoscape software. Nodes represent genes and lines indicate protein interactions between genes. **(B)** Center gene map. The redder the color of the nodes, the higher the degree is.

We built a PPI network of the SDR gene family in *A. phaeospermum* with 29 nodes and 172 edges (Figure 4A). The larger diameter of the protein node icon represents the larger degree, and the thicker interaction lines between the two proteins represent the higher interaction score. Proteins ApSDR53C2 and ApSDR548U2 were identified as the two interaction centers of the two interaction networks.

The cytoHubba plugin of Cytoscape was used to identify the top 10 hub genes of network node degree in the PPI network in accordance with the Degree method (Figure 4B). The degree of a protein node represents the number of proteins interacting with it. The higher the degree, the higher the importance of the protein in the organism, which is essential to maintain its life activities, and also essential to maintain the morphology of the whole network. The analysis yielded five proteins in the constructed network with a degree higher than 10. The highest one was ApSDR548U2 with a degree of 13, followed by the protein ApSDR53C2 with a degree of 12. Among the top 10 hub genes with network node degree, seven genes belonged to the Classical class, two genes belonged to the Unassigned class and one belonged to the Extended class.

### Analysis of SDR gene expression pattern in different infestation periods and different stresses of *A. phaeospermum*

**FIGURE 5:**
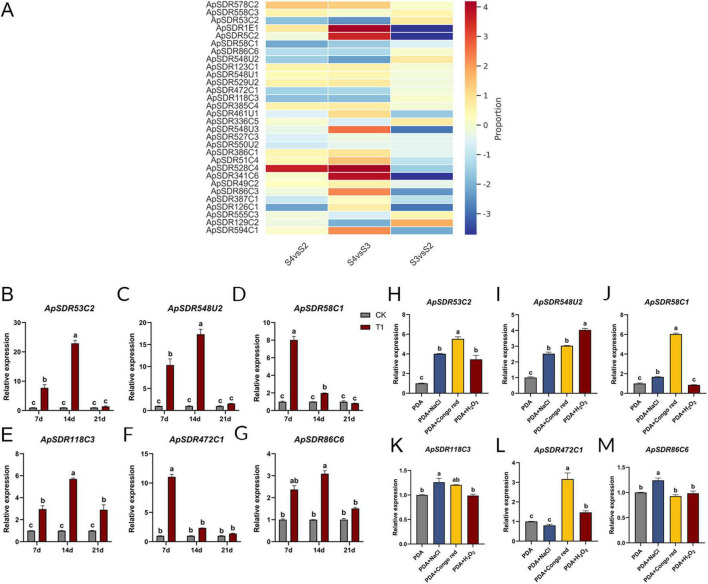
**(A)** Heatmap of the top 30 expressed *ApSDRs* in the transcriptome sequencing data. Proportion represents the log2 value of the fold change in the expression of *ApSDRs* between different periods. Red represents an up-regulation of gene expression and blue represents a decrease in gene expression. The rows represent transcription units. **(B–G)** Expression levels of the six candidate *ApSDRs* at different infestation times were determined by qPCR. Tubulin from *A. phaeospermum* was used as an internal reference gene. CK: *A. phaeospermum* inoculated in a PDA medium coated with 200 μL of sterile deionized water. T1: *A. phaeospermum* inoculated in a PDA medium coated with 200 μL of sterile hybrid bamboo tissue fluid. **(H–M)** Expression levels of the six candidate *ApSDRs* under stress conditions were determined by qPCR. Tubulin from *A. phaeospermum* was used as an internal reference gene. There are significant differences in the relative expression of different lowercase letters (*p* < 0.05).

The expression data of all *ApSDRs* were collected from the transcriptome sequencing data of *A. phaeospermum* infesting *B. pervariabilis × D. grandis* at 0 (S1 period: uninfested phase), 7 (S2 period: contact phase), 14 (S3 period: penetration phase), and 21days (S4 period: incubation phase). The previous study showed that *A. phaeospermum* infested hybrid bamboos in the contact and penetration phase (S2 and S3), which caused only PAMP-triggered immunity (PTI) response in hybrid bamboos, while the effector-triggered immunity (ETI) response of hybrid bamboos was activated in the incubation phase (S4) (Fang et al., 2022). Considering the traits of hybrid bamboos, like their thick cuticle and high fiber content in their branches, it was hypothesized that the genes which were significantly up-regulated during S2 and S3 infestation and down-regulated during S4 may be the key pathogenicity factors for the invasion of hybrid bamboos. As shown in Figure 5A, among the top 30 *ApSDRs* in expression, *ApSDR53C2*, *ApSDR58C1*, *ApSDR86C6*, *ApSDR548U2*, *ApSDR472C1*, *ApSDR118C3* were significantly up-regulated during S2 and S3 infestation and down-regulated during S4.

Both transmembrane proteins and secreted proteins have signal peptides. In addition, transmembrane proteins should have transmembrane structures and hydrophobic regions. As shown in Supplementary Figure S4A, in the signal peptide analysis by signalP, only proteins ApSDR53C2 and ApSDR58C1 were present with the signal peptides. In the transmembrane region analysis by TMHMM, proteins ApSDR53C2, ApSDR58C1, and ApSDR86C6 were present in the transmembrane regions (Supplementary Figure S4B). The hydrophobicity of amino acids reflects the folding of proteins, with hydrophobic regions occurring in potential transmembrane regions and playing an important role in maintaining the tertiary structure of proteins, which includes biological membrane structure. Hydropathicity/hydrophobicity mapping of proteins can provide a reference for the identification of transmembrane regions of proteins. In general, the transmembrane region of a protein is hydrophobic, while the portion outside the membrane is hydrophilic. Hydrophobic proteins have a disproportionately high number of hydrophobic amino acid residues throughout the polypeptide chain compared to hydrophilic ones, and hydrophilic proteins have the opposite pattern. As shown in Supplementary Figure S4C, the protein hydropathicity/hydrophobicity maps of six candidate ApSDRs were plotted by ProtScale. The other four proteins were hydrophilic proteins, while proteins ApSDR53C2 and ApSDR58C1 were hydrophobic proteins, and their predicted results of the hydrophobic region matched the predicted results of the transmembrane region. It is hypothesized that proteins ApSDR53C2 and ApSDR58C1 are transmembrane proteins.

Six putative ApSDRs’ protein tertiary structures were estimated using SWISS-MODEL (Supplementary Figure S4D). According to the prediction results, all six proteins have the conserved ‘Rossmann-fold’ three-dimensional structure of the SDR family, which includes the catalytic and cofactor-binding sites (Kavanagh et al., 2008). The “Rossmann-fold” is composed of three α-helices on each side and a center β-fold with 6–7 strands.

As can be shown in Figures 5B–G, after inoculating *A. phaeospermum* into PDA mediums containing hybrid bamboo tissue fluid, the expression of all six genes dramatically dropped at day 21, and it exceeded that of the control at days 7 and 14. Among these, *ApSDR58C1* and *ApSDR472C1* gene expression was considerably higher on day 7 compared to the other two periods and exhibited a declining trend from days 7 to 21. Between days 7 and 14, the expression of the genes *ApSDR53C2*, *ApSDR548U2*, *ApSDR86C6*, and *ApSDR118C3* increased at day 14 and then decreased at day 21. The expression of *ApSDR53C2* and *ApSDR548U2* was substantially higher than that of the other genes. This appears to suggest that the six SDR genes’ expression rose when *A. phaeospermum* was infested with *B. pervariabilis* × *D. grandis*. These genes may be crucial to the infestation process, with *ApSDR53C2* and *ApSDR548U2* having a more substantial impact. The transcriptome sequencing of *A. phaeospermum* infesting *B. pervariabilis* × *D. grandis* revealed the same trends in the expression of these six genes.

As shown in Figures 5H–M, in the PDA medium with the addition of 2 mol/L NaCl, the expression levels of the other five genes were considerably greater than that in the unstressed control, except for gene *ApSDR472C1*. With the exception of *ApSDR86C6*, the expression levels of the other five genes were considerably higher under the condition of 2 mg/mL Congo red than in the unstressed control. Under the treatment of 40 mmol/mL H_2_O_2_, only *ApSDR53C2* and *ApSDR548U2* genes were appreciably highly expressed compared to those in normal PDA medium, while the other four genes showed no significant changes. Nearly all of these six genes’ expression levels increased significantly under different stressors, particularly in Congo red stress and NaCl stress.

Out of the six genes, *ApSDR53C2* and *ApSDR548U2* exhibited the greatest variation in expression from the control under various stress scenarios, potentially indicating a crucial function in the pathogenicity exhibited by the pathogens.

### Phenotypic analysis of SDR deletion mutants

The knockout vectors for *ApSDR53C2* and *ApSDR548U2* were constructed, which were successfully assayed by an improved isolation marker method (Supplementary Figure S5). A large number of transparent rounded protoplasts of *A. phaeospermum* were obtained after centrifugal washing (Supplementary Figure S6). Recombinant vectors were transformed by protoplasts separately to 350 μg/mL of hygromycin in TB3. As shown in Supplementary Figure S7A, colonies grown under 350 μg/mL of hygromycin were inoculated onto PDA supplemented with 400 μg/mL of hygromycin, and the colonies grown under 400 μg/mL of hygromycin were positive knock-out transformants. The method of obtaining backfill strains is the same as above. The positive complemented transformants were shown in Supplementary Figure S7B. Then, “hph,” “*ApSDR53C2*,” “*ApSDR548U2*,” “*ApSDR53C2*-5-hph,” “*ApSDR53C2*-3-hph,” “*ApSDR548U2*-5-hph,” “kanMx” fragments of positive transformant strains were identified by PCR, as Supplementary Figure S8 illustrates. Knock-out mutants Δ*ApSDR53C2*, Δ*ApSDR548U2*, and co-knockout mutant Δ*ApSDR53C2*:*ApSDR548U2*, were effectively obtained for both genes and back-complemented with three deletion mutants.

**FIGURE 6:**
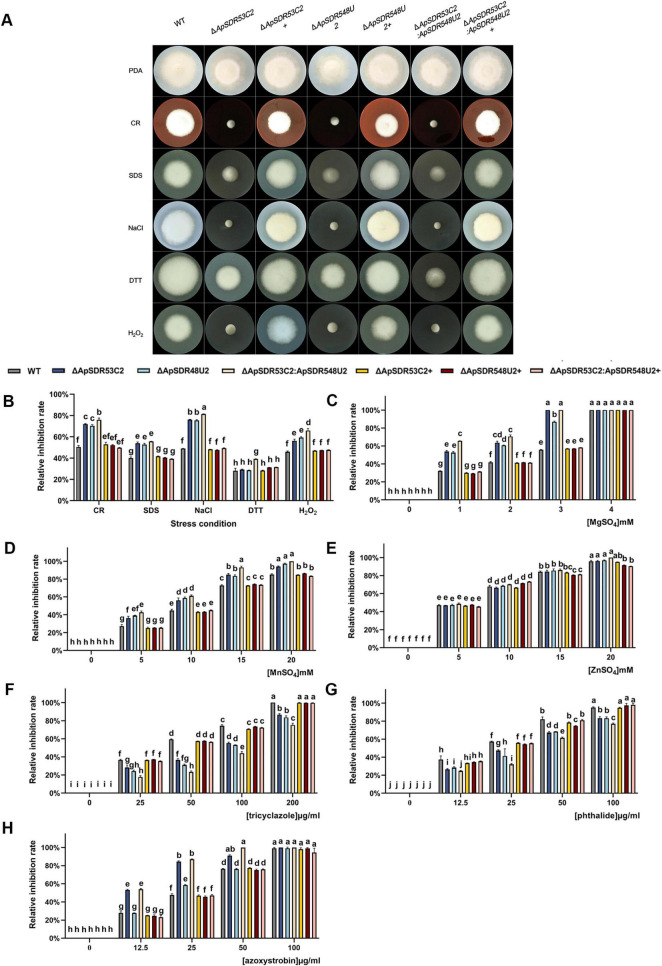
Differences among wild-type (WT), deletion mutant, and back mutant strains under different stresses. **(A)** Comparison of colony morphology and resistance of WT, deletion mutant, and backfill mutant strains. Note: Inoculation of WT, deletion mutants (Δ*ApSDR53C2*, Δ*ApSDR548U2*, and Δ*ApSDR53C2*:*ApSDR548U2*), and back-complementary mutants (Δ*ApSDR53C2*+, Δ*ApSDR548U2*+, and Δ*ApSDR53C2*:*ApSDR548U2*+) was carried out in PDA medium or PDA medium supplemented with various stressors for 5 days at 25°C. **(B–H)** Bar graphs showing WT, deletion mutant, and backfill mutant strains in the relative inhibition rates after 5 days of growth on PDA mediums supplemented with stress reagents (CR, SDS, NaCl, DTT, and H_2_O_2_), heavy metals (Mg^2+^, Mn^2+^, and Zn^2+^), and fungicides (tricyclazole, phthalide, and azoxystrobin). There are significant differences in the relative inhibition of different lowercase letters (*p* < 0.05).

As shown in Figures 6A,B, compared to the WT and backfilled strain, the deletion mutants’ phenotypes and relative inhibition rate were noticeably different. For the cell wall stress sensitivity assay, the relative inhibition rate of Δ*ApSDR53C2*, Δ*ApSDR548U2*, and Δ*ApSDR53C2*:*ApSDR548U2* colonies after the addition of 2 mg/mL Congo red was 71.9, 70.3, and 75.9%, respectively, and all three mutant strains were more particularly responsive to Congo red stress than the WT strain (50.4%). The relative inhibition of Δ*ApSDR53C2*, Δ*ApSDR548U2*, and Δ*ApSDR53C2*:*ApSDR548U2* colonies after the addition of 0.01% SDS was 53.9, 52.7, and 55.7%, respectively, and all three mutant strains were more particularly responsive to SDS stress than the WT strain (40.0%). Comparing the difference in sensitivity to osmotic stress between the transformant strains and the WT strain, the relative inhibition of Δ*ApSDR53C2*, Δ*ApSDR548U2*, and Δ*ApSDR53C2*:*ApSDR548U2* colonies was considerably greater than WT strain (49.0%) under 2 mol/L NaCl treatment, and the relative growth inhibition of the three deletion mutants was 76.0, 75.4, and 81.3%, respectively. In addition, in the oxidative stress sensitivity assay, the three mutants showed significantly lower tolerance to H_2_O_2_. The growth area of Δ*ApSDR53C2*, Δ*ApSDR548U2*, and Δ*ApSDR53C2*:*ApSDR548U2* colonies was significantly reduced by 56.2, 59.3, and 65.8%, respectively, at a concentration of 40 mmol/L H_2_O_2_. Nevertheless, none of the three knockout mutants’ colony growth was profoundly impacted by the 5 mmol/L DTT stress. The phenotypes of the WT and complemented strains did not differ significantly. These results suggest that *ApSDR53C2* and *ApSDR548U2* have important roles in cell wall inhibitor stress response, osmotic stress response, and oxidative stress response of *A. phaeospermum*, without participating in the endoplasmic reticulum stress of its cells themselves.

Comparing the heavy metal stress of the respective knockout transformant strains of the *ApSDR53C2* and *ApSDR548U2* genes, their co-knockout transformant strain, and the backfill strains with the WT strains (Figures 6C–E), the relative inhibition of Δ*ApSDR53C2*, Δ*ApSDR548U2*, and Δ*ApSDR53C2*:*ApSDR548U2* colonies on PDA mediums with added Mg^2+^ and Mn^2+^ was significantly increased compared to that of the WT strain, and the knockout transformants showed a greater degree of increase in the relative inhibition with the addition of Mg^2+^. The differences in the relative inhibition of Δ*ApSDR53C2*, Δ*ApSDR548U2*, and Δ*ApSDR53C2*:*ApSDR548U2* versus the WT strain were not significant with Zn^2+^ added. Under the three heavy metal stresses, the relative inhibition of the backfilled strains and the WT strain did not differ significantly. It suggests that the deletion of these two genes resulted in a decrease in resistance to Mg^2+^ and Mn^2+^ in *A. phaeospermum*, and thus Mg^2+^ and Mn^2+^ may promote the enzymatic activities of the proteins encoded by *ApSDR53C2* and *ApSDR548U2*.

Comparing the fungicide stress of the transformant strains with the WT strains (Figures 6F–H), Δ*ApSDR53C2*, Δ*ApSDR548U2*, and Δ*ApSDR53C2*:*ApSDR548U2* strains showed a considerable decrease in their fungicide stresses under the anti-osmotic fungicide tricyclazole and phthalide, the relative inhibition rate was considerably decreased compared to the WT strain, where the difference in relative inhibition rate was more significant under the stress of tricyclazole. Under azoxystrobin treatment, Δ*ApSDR53C2* and Δ*ApSDR53C2*:*ApSDR548U2* strains showed a significant difference in relative inhibition rate compared to the WT strain, but the Δ*ApSDR548U2* strain did not. Under the three fungicide treatments, the relative inhibition of the backfilled strains and the WT strain did not differ significantly.

### Pathogenicity analysis of SDR knockout mutants

**FIGURE 7:**
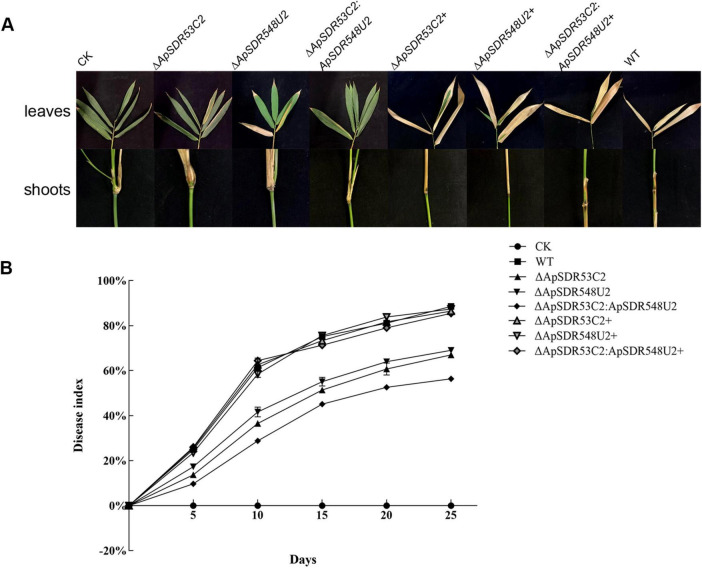
Pathogenicity analysis of mutants. **(A)** Symptoms of *B. pervariabilis* × *D. grandis* leaves and branches inoculated for 25 days with sterile water, mycelial suspensions of deletion mutants, backfill mutants, and WT strains. **(B)** Dynamics of disease index of *B. pervariabilis* × *D. grandis* infected by different strains. CK, Δ*ApSDR53C2*, Δ*ApSDR548U2*, Δ*ApSDR53C2*:*ApSDR548U2*, Δ*ApSDR53C2*+, Δ*ApSDR548U2*+, and Δ*ApSDR53C2*:*ApSDR548U2*+, and WT represent changes in the disease indices of *B. pervariabilis* × *D. grandis* infected by the corresponding strains. All determinations were repeated three times and data were analyzed using one-way ANOVA and Duncan’s range test in SPSS 16.0.

Mycelial suspension from WT and the corresponding mutants were prepared and inoculated on the shoots and leaves of *B. pervariabilis × D. grandis* separately to perform a pathogenicity test. The WT strain was shown to generate much more severe symptoms than the Δ*ApSDR53C2*, Δ*ApSDR548U2*, and Δ*ApSDR53C2*:*ApSDR548U2* mutants (Figure 7A).

Figure 7B displays the quantitative measurements of the disease indices of hybrid bamboo inoculated with various strains. The disease indices of hybrid bamboos rose with increasing inoculation time, regardless of whether they were inoculated with the WT, Δ*ApSDR53C2*, Δ*ApSDR548U2*, or Δ*ApSDR53C2*:*ApSDR548U2* mutant. After 25 days, the disease indices of them reached 83.53, 53, 55, and 50%, while that of hybrid bamboo inoculated with sterile water was 0. We also found that the disease index of hybrid bamboos inoculated with WT at the same time was significantly higher than that of all three deletion mutants, whereas that of the Δ*ApSDR53C2* mutant was lower than the Δ*ApSDR548U2* mutant, the Δ*ApSDR53C2*:*ApSDR548U2* mutant was lower than that of the Δ*ApSDR53C2* and Δ*ApSDR548U2* mutants. There was no discernible difference between the disease indices of hybrid bamboos inoculated with all backfill mutants and the WT. This result suggests that genes *ApSDR53C2* and *ApSDR548U2* play important roles in *A. phaeospermum* virulence, and the gene *ApSDR53C2* has a stronger virulence role.

### The function of ApSDR53C2 in regulating melanin biosynthesis and cellulose penetration processes

**FIGURE 8:**
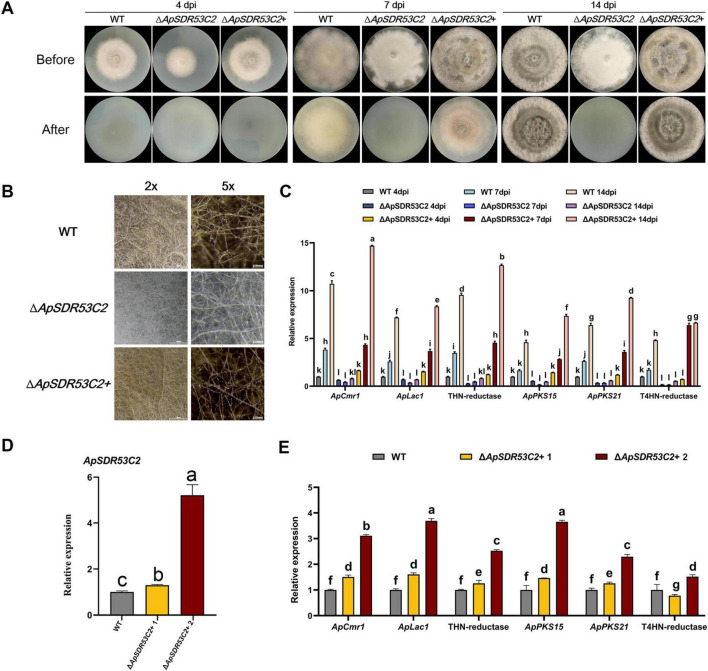
Analysis of *ApSDR53C2* regarding melanin biosynthesis and cellulose membrane penetration functions. **(A)** Analysis of the cellulose membrane penetration ability of wild type, Δ*ApSDR53C2* and Δ*ApSDR53C2*+. **(B)** Stereomicroscope observation of melanized hyphae. **(C)** The qPCR detection of melanin synthesis-related gene expression levels at different growth periods. **(D)** Various backfill transformants with differences in *ApSDR53C2* expression. **(E)** Variations in melanin synthesis levels at different *ApSDR53C2* expression levels. There are significant differences in the relative expression of different lowercase letters (*p* < 0.05).

As shown in Figure 8A, after cellulose membrane treatment of the strains to be analyzed for 7 days, the WT strains were able to penetrate the cellulose membrane, However, Δ*ApSDR53C2* was not able to pass through the cellulose membrane, and no obvious hyphae were observed in the PDA mediums after uncovering the membranes. The level of Δ*ApSDR53C2*+ cellulose membrane penetration was close to that of the WT.

After 14 days of cellulose membrane induction, the WT, Δ*ApSDR53C2*+ colonies were obviously blackened, while the Δ*ApSDR53C2* colonies were lighter in color (Figure 8B), which indicated that *ApSDR53C2* has the function of regulating melanin synthesis. The expression of melanin synthesis-related genes was all considerably lower in the Δ*ApSDR53C2* than in the WT strain, whereas the expression in the gene-function backfilling strains was close to that of the WT (Figure 8C). Prolonging the cellulose membrane induction time, the expression levels of melanin synthesis-related genes in the Δ*ApSDR53C2* still could not be induced to rise to the WT level. The aforementioned findings suggest that *ApSDR53C2* influences the level of melanin synthesis during the penetration of cellulose membranes, and the process of cellulose membrane penetration induces the production of melanin in *A. phaeospermum*, suggesting that *ApSDR53C2* promotes mycelial penetration by influencing melanin synthesis.

Two *ApSDR53C2* complemented mutants with widely varying *ApSDR53C2* gene expression were chosen, with the aim of exploring the changes in melanin synthesis-related genes’ expression level with different *ApSDR53C2* gene expression and further demonstrating the correlation between the *ApSDR53C2* gene and melanin biosynthesis. The *ApSDR53C2* expression in Δ*ApSDR53C2* complemented mutants was analyzed under cellulose membrane-induced conditions. The results of qPCR demonstrated that *ApSDR53C2* expression levels in Δ*ApSDR53C2*+ 1 and Δ*ApSDR53C2*+ 2 were 1.30-fold and 5.67-fold higher than those in the WT (Figure 8D). Detecting the melanin synthesis-related genes’ expression (Figure 8E), the expression levels in Δ*ApSDR53C2*+ 2 were significantly higher than those of Δ*ApSDR53C2*+ 1. It indicated that the *ApSDR53C2* gene level was positively correlated with melanin synthesis-related genes, and positively promoted the biosynthesis of melanin in *A. phaeospermum*.

**FIGURE 9:**
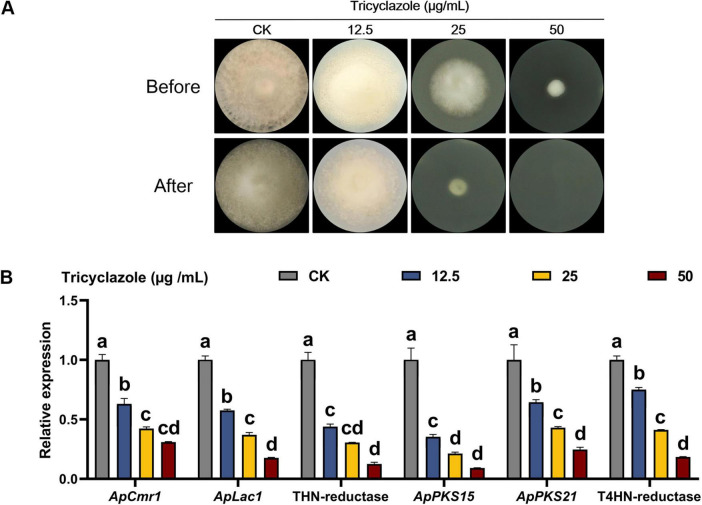
**(A)** Analysis of cellulose membrane penetration in tricyclazole-treated strains of *A. phaeospermum.* Before: After 7 days of inoculation and growth on PDA medium covered with a sterile cellulose membrane, the strains were examined for morphology before the membrane was removed. After: The strains were cultivated for 7 days to be examined in morphology after the cellulose membrane was removed. **(B)** Tricyclazole-treated strains were examined for melanin synthesis-related gene expression levels. There are significant differences in the relative expression of different lowercase letters (*p* < 0.05).

The effect of melanin on the penetration of cellulose membranes by *A. phaeospermum* was analyzed using the melanin synthesis inhibitor tricyclazole (Figure 9A). The findings demonstrated that tricyclazole could effectively prevent *A. phaeospermum* from penetrating the cellulose membrane, and that when the concentration of tricyclazole increased, the expression of genes linked to melanin formation in the treated group declined dramatically (Figure 9B). This suggests that melanin is crucial for *A. phaeospermum*’s ability to penetrate the cellulose membrane.

## Discussion

Widely distributed in eukaryotes, the SDR family is a gene superfamily that is challenging to identify due to its low sequence similarity among members and conserved “Rossmann-fold” three-dimensional structure. Few studies have been conducted on the bioinformatic identification and analysis of the SDR gene family, which has only been systematically identified in species such as *Oreochromis niloticus* (Zhang et al., 2021) and *Medicago truncatula* (Yu et al., 2021). Additionally, there aren’t many studies that are pertinent to fungi. 213 SDR genes were identified in the *M. truncatula* genome and 119 SDR genes in the O. niloticus genome. We systematically identified and screened 180 SDRs in *A. phaeospermum* using blast, HMM model, SMART, and NCBI CDD. In line with the findings of the investigations by Gabrielli et al. (2022) and Kallberg et al. (2002), the analysis showed that the number of introns in different groups of 180 *ApSDRs* differed significantly, and it was hypothesized that the functions of SDRs would differ among groups. *ApSDRs* were irregularly distributed on 18 of the 19 chromosomes of *A. phaeospermum*, suggesting that the arrangement of *ApSDRs* on every chromosome were uneven, which is in line with the results of chromosomal distribution in *Medicago truncatula* (Yu et al., 2021). In addition, the highest number of homologous SDR genes was found between *A. phaeospermum* and *Arthrinium hydei* of the same genus, with a total of 141 pairs of homologous genes identified, and the lowest number of SDR homologous pairs was found between the species of *Fusarium oxysporum* of different genera and families, which suggests that the number of homologous pairs of SDR genes varies according to the distance of the kinship between species, and that the closer the kinship is, the higher the number of homologous pairs of SDR genes. The closer the affinity, the larger the number of SDR homologous pairs.

Invasion of pathogens triggers two types of immune systems: PTI and ETI, and the PTI response is the first level of immune response of plants against pathogen infestation (Yu et al., 2024). Based on the transcriptome sequencing results of various disease stages of *A. phaeospermum* infestation of *B. pervariabilis × D. grandis*, the expression of the *ApSDRs* detected bioinformatically was higher, with the expression of *ApSDR53C2*, *ApSDR58C1*, *ApSDR86C6*, *ApSDR548U2*, *ApSDR472C1*, and *ApSDR118C3* significantly up-regulated in the contact and penetration phase, and down-regulated in the incubation phase. These six genes were thought to be the main pathogenicity factors for *A. phaeospermum*’s invasion of hybrid bamboo. Thus, qPCR experiments were conducted, finding that the expression levels of *ApSDR53C2* and *ApSDR548U2* were more significant than the others during the process.

The next results confirmed that the knockout and co-knockout mutants of *ApSDR53C2*, *ApSDR548U2* showed a considerable increase in response to cell wall disturbing stress, hyperosmotic stress, extracellular oxidative stress, and Mg^2+^ and Mn^2+^ stresses, which were restored to a level similar to that of the WT after backfilling. This implies that the response of *A. phaeospermum* to a variety of unfavorable external conditions is considerably influenced by these two *ApSDRs*, causing the cell wall structure of the knockout transformants to be altered and become more sensitive to Congo red. The deletion of these two genes led to a decrease in Mg*^2+^* and Mn*^2+^* resistance in *A. phaeospermum*, thus Mg^2+^ and Mn^2+^ may promote the enzymatic activity of the proteins encoded by *ApSDR53C2* and *ApSDR548U2*, which is in agreement with the finding of SDR-X1 in *Pseudomonas citronellae* (Zheng et al., 2016). The mechanism of genes *ApSDR53C2* and *ApSDR548U2* in osmotic fungicide resistance (tricyclazole and phthalide) was further explored, and it was found that deletion of these two genes in the transformants showed a considerable decrease in relative inhibition compared to the WT strains under the stress of tricyclazole and phthalide. It indicates that both genes are targeted and repressed by osmotic resistant fungicides. The mechanism of action of tricyclazole and phthalide fungicides is the inhibition of melanin production in appressorium, and SDRs are closely related to melanin biosynthesis, so it was hypothesized that the genes *ApSDR53C2* and *ApSDR53C2* might regulate melanin biosynthesis in *A. phaeospermum*.

Moreover, the knockout backfill studies eliminated epigenetic modifications brought on by the mutagenicity of protoplast transformation itself, and the pathogenicity of the mutant strains with the *ApSDR53C2* and *ApSDR548U2* genes knocked out was considerably lower than the WT. This result showed that all three deletion mutant strains, Δ*ApSDR53C2*, Δ*ApSDR548U2*, and Δ*ApSDR53C2*:*ApSDR548U2*, were significantly less virulent than the WT strain, and in particular, the Δ*ApSDR53C2* mutant. Thus, the *ApSDR53C2* and *ApSDR548U2* genes may be associated with *A. phaeospermum* virulence and *ApSDR53C2* plays a more critical role. This speculation is consistent with the conclusion that *ApSDRs* have an important role in virulence (Kwon et al., 2010). The biological functions of SDRs are diverse and include multifunctionality and fundamental importance in metabolic processes as well as important roles in cellular differentiation, and signal transduction (Kallberg et al., 2002; Persson et al., 2009). In line with the findings of this study, which indicate that particular *ApSDRs* play considerable roles in pathogenicity, it has been demonstrated that the SDR gene family member 3HNR is a crucial gene for pathogenicity in *M. oryzae* (Kwon et al., 2010). The role of *ApSDRs* in growth and development, stress response, and infestation was determined in this study, which is consistent with the findings that *MoSDR1* is a key metabolic enzyme regulating the development and pathogenicity of *M. oryzae* (Sesma and Osbourn, 2004). Our results will provide new insights for SDR gene family identification and functional studies in other species.

In order to explore the pathogenicity mechanism of *ApSDR53C2*, this study analyzed the function of *ApSDR53C2* in regulating the cellulose membrane of *A. phaeospermum* infestation by cellulose membrane penetration assay, finding that *A. phaeospermum* began to possess the ability of cellulose membrane penetration after reaching the contact and penetration phases, which were characterized by a significant increase in the expression of *ApSDR53C2*. According to qPCR experiments, there was a positive correlation between the level of melanin biosynthesis and the expression level of *ApSDR53C2*, meaning that melanin production was enhanced by raising the transcription level of the *ApSDR53C2* gene. In the early stages of pathogenesis, melanin controls the infiltration of the host epidermis by fungal pathogens. The process of infiltration filaments formed by the appressorium to penetrate the plant epidermis depends on the maintenance of the appressorium’s strong osmotic pressure, which is dependent on the melanin deposited inside its cell wall (Day, 1998). In the study of the infestation pattern of *A. phaeospermum*, it was found that the conidia in the mycelium and ascospores of *A. phaeospermum* are the most important form of overwintering and the most important source of primary infestation for the development of the disease. Conidia sprout bud tubes and then form appressorium at the tip, and mature appressorium specializes in mycelium from the bottom to invade host cells (Li et al., 2013). However, the relationship between melanin and appressorium function in *A. phaeospermum* is not clear. We examined the connection between the capacity of cellulose membrane penetration at various growth stages and the expression of genes linked to melanin formation, and found that *A. phaeospermum* began to have that ability after a significant increase in the expression of melanin synthesis-related genes. In this study, we analyzed the effect of melanin on cellulose membrane penetration of *A. phaeospermum* using melanin synthesis inhibitors. The results showed that the melanin synthesis inhibitor, tricyclazole, significantly reduced the level of melanin synthesis in *A. phaeospermum*, which in turn inhibited cellulose membrane penetration. Thus, the process of cellulose membrane penetration by *A. phaeospermum* requires melanin accumulation, consistent with the results that melanin accumulation is required to penetrate the maize epidermis during infestation of *Setosphaeria turcica* (Guo et al., 2022).

In summary, the genes *ApSDR53C2* and *ApSDR548U* contribute to *A. phaeospermum*’s ability to withstand hyperosmotic stress, and extracellular oxidative stress, as well as being important virulence factors in *A. phaeospermum*, which can promote melanin biosynthesis in order to facilitate the penetration of appressorium into the cell wall of the hybrid bamboo during the contact and penetration phases and produce pathogenicity. The work offers helpful relevant information for further research on the biological roles of *ApSDRs*, particularly with regard to the preservation of osmotic pressure in the appressorium. In order to establish a new foundation for the pathogenic mechanism of *A. phaeospermum*. The next challenge will be to reveal the signaling mechanism of *ApSDRs* involved in melanin biosynthesis, providing a new basis for the pathogenic mechanism of *A. phaeospermum*.

## Data Availability

The genome sequence of *A. phaeospermum* can be found in the NCBI GenBank under the accession number QYRS00000000. The transcriptome sequencing data of *A. phaeospermum* infesting *B. pervariabilis* × *D. grandis* in this study can be found in the NCBI Sequence Read Archive (SRA) under the accession number SAMN19312317.

## Footnotes

1
https://www.ncbi.nlm.nih.gov/

2
http://hmmer.org/

3
https://www.ncbi.nlm.nih.gov/cdd/

4
http://smart.embl-heidelberg.de/

5
http://www.megasoftware.net/

6
http://meme-suite.org/tools/meme

7
http://gsds.cbi.pku.edu.cn/

8
http://mg2c.iask.in/mg2c_v2.1/

9
https://orthomcl.org/orthomcl/

10
http://chibba.pgml.uga.edu/mcscan2

11
https://www.omicshare.com/tools/Home/Soft/splitviolin

12
https://cn.string-db.org/

13
https://services.healthtech.dtu.dk/services/SignalP-6.0/

14
https://services.healthtech.dtu.dk/services/TMHMM-2.0/

15
https://web.expasy.org/protscale/

16
https://www.swissmodel.expasy.org/
