# Supplementary material for: Genome-wide identification of short-chain dehydrogenases/reductases genes and functional characterization of ApSDR53C2 in melanin biosynthesis in Arthrinium phaeospermum
Source: Front Microbiol. 2025 Jan 30;16:1532162. doi: 10.3389/fmicb.2025.1532162 (PMC11821928; doi:10.3389/fmicb.2025.1532162)
Supplement: Supplementary file 1 [file Data_Sheet_1.docx]

Supplementary Material


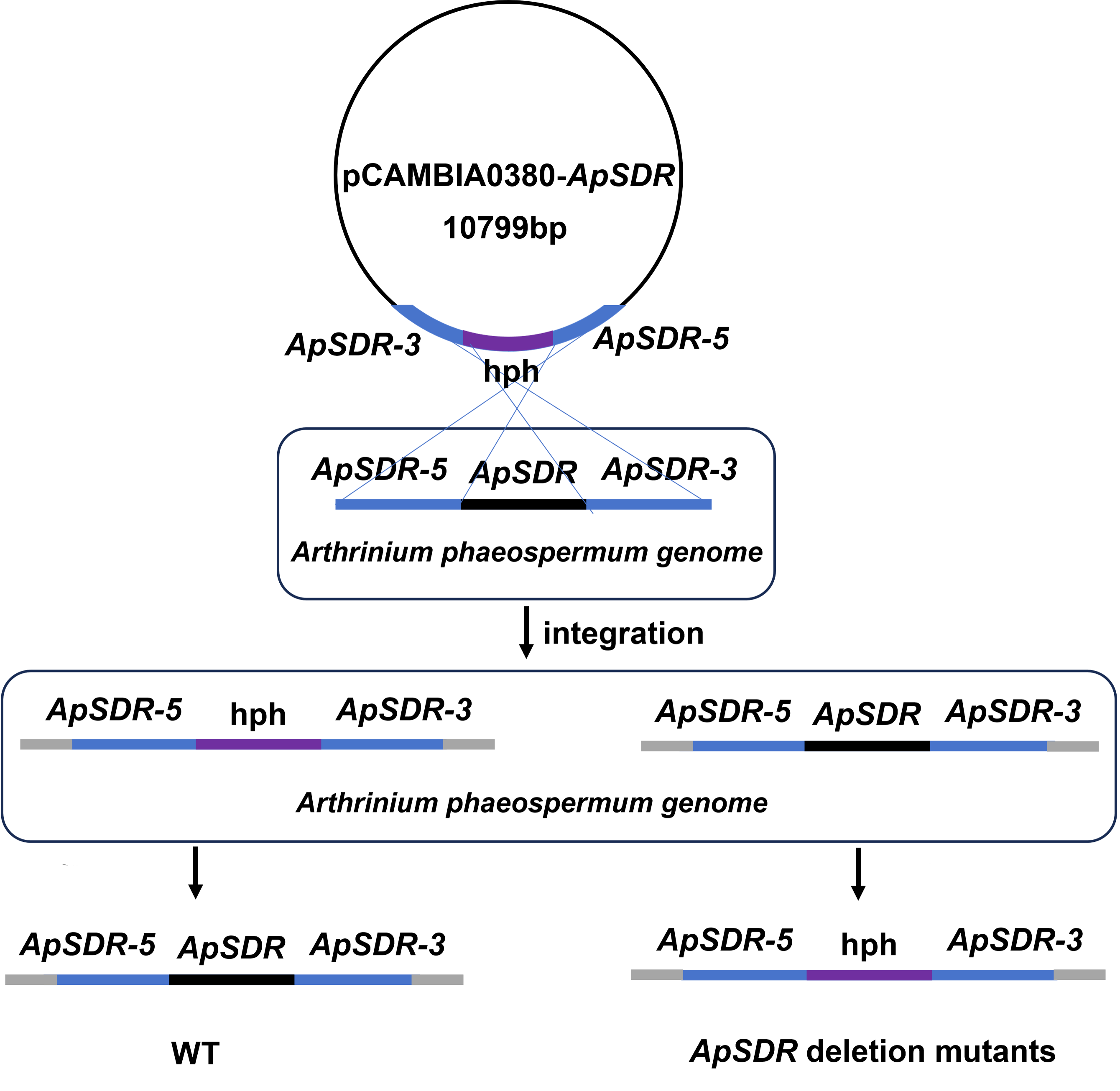


**Figure S1.** Strategy employed in this study for the knockout of SDR gene.


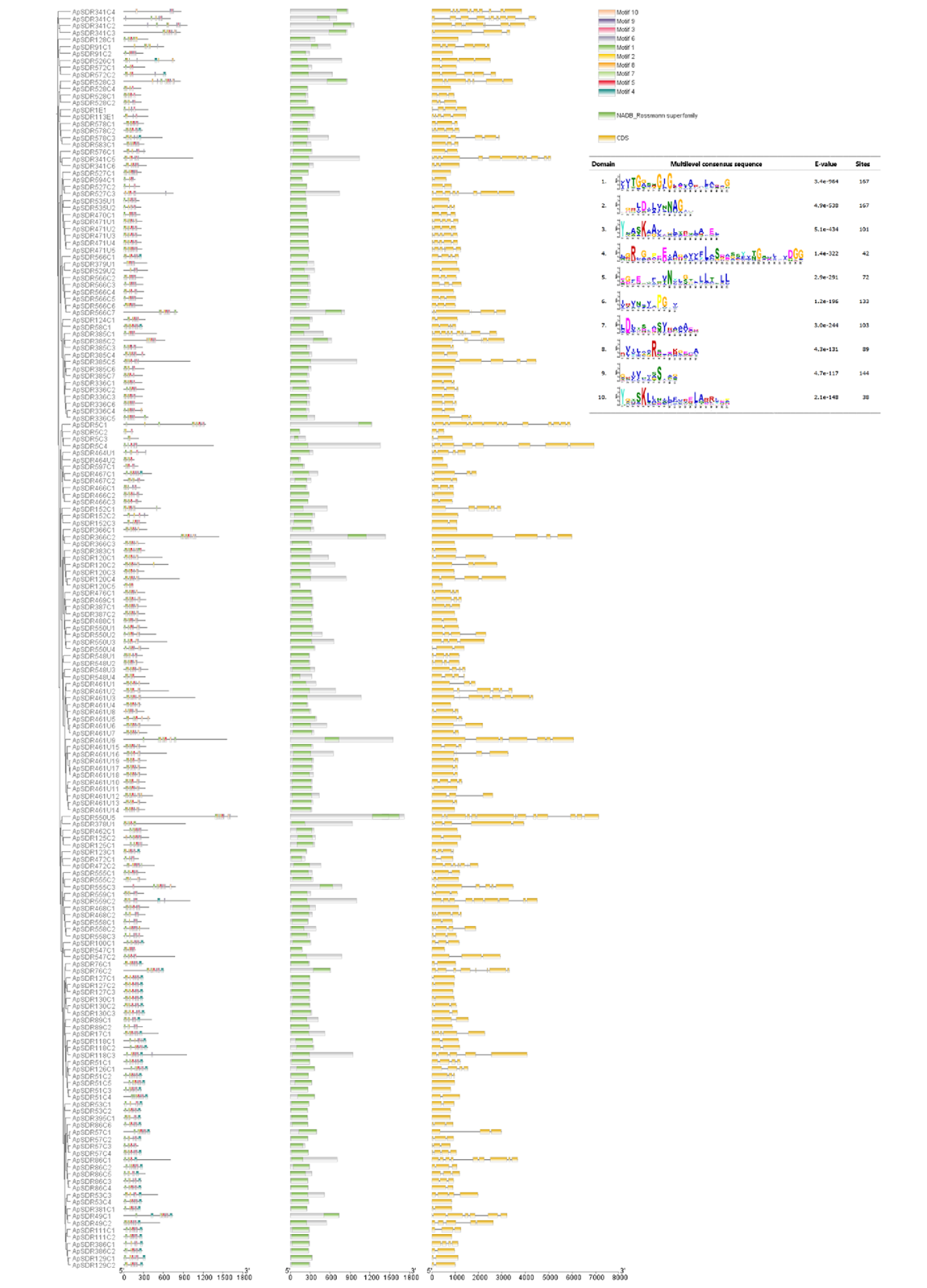


**Figure S2.** Analysis of conserved motifs, conserved structural domains, and gene structures of *ApSDRs*. The small panel on the right is the colored boxes, which displayed the size and multilevel consensus sequence of motifs 1 to 10.


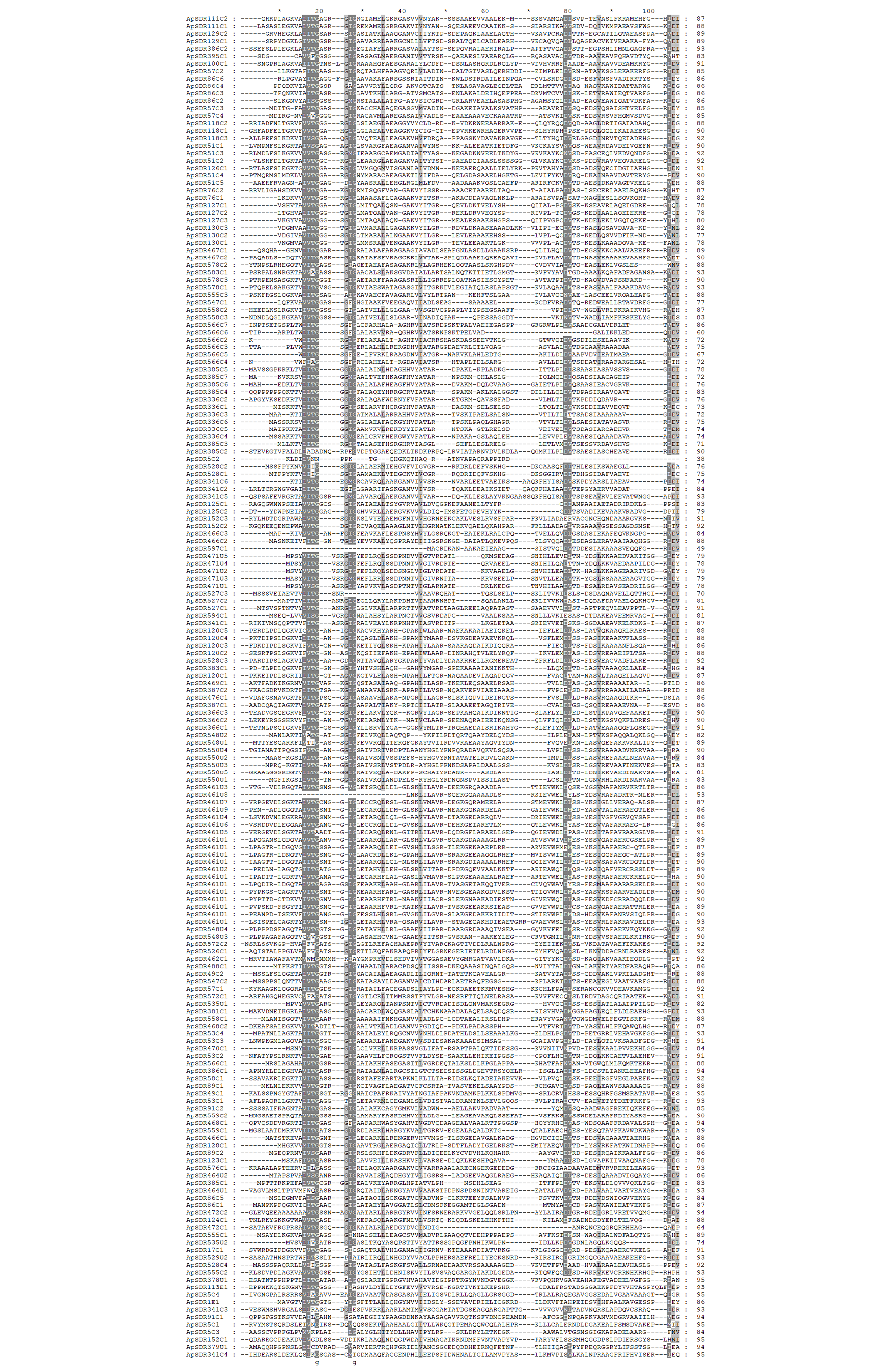


**Figure S3.** Multiple sequence comparison analysis of *ApSDRs*. Dark gray and light gray grayscale values are >80% and >60%, respectively.


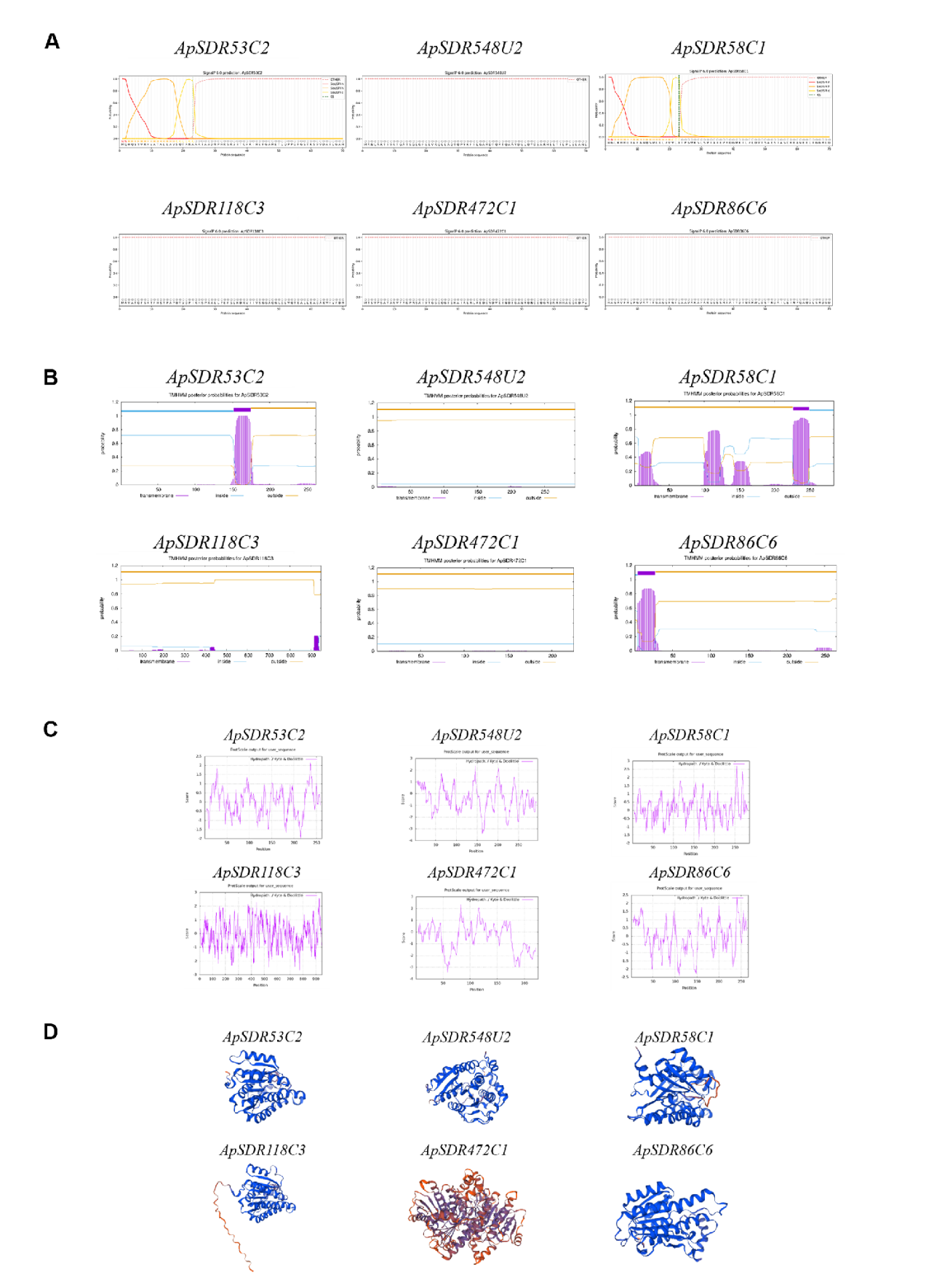


**Figure S4.** (A) Signal peptide prediction of six candidate ApSDR proteins. (B) Transmembrane structure prediction of six candidate ApSDR proteins. (C) Protein hydropathicity/hydrophobicity maps of six candidate ApSDR proteins. (D) Protein tertiary structure prediction of six candidate ApSDR proteins.

**
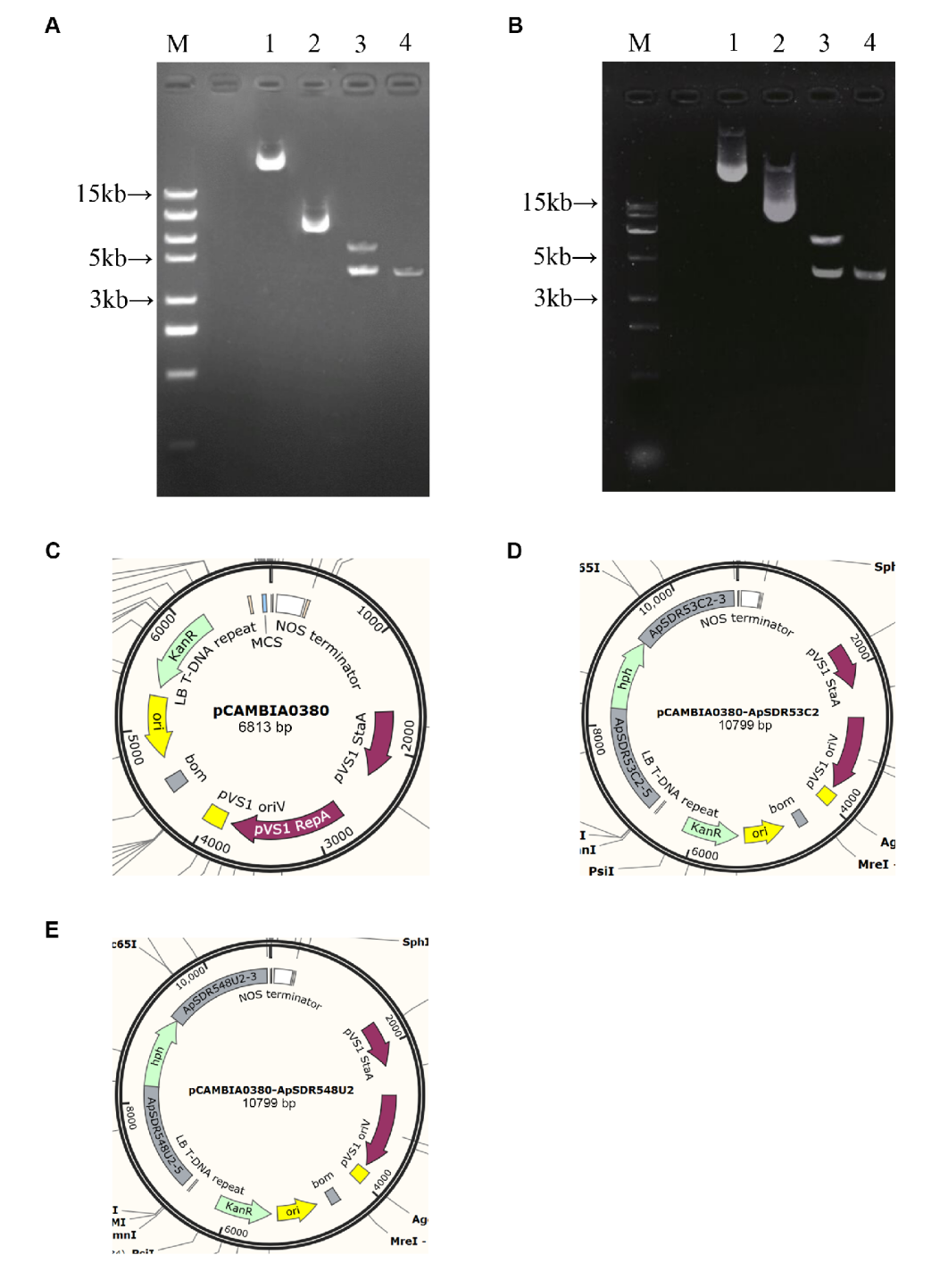
**

**Figure S5.** Knockdown vectors pCAMBIA0380-*ApSDR53C2* and pCAMBIA0380-*ApSDR548U2* were enzymatically verified. (A) Knockdown vectors pCAMBIA0380-*ApSDR53C2* were enzymatically verified. Note: M: DL15000 DNA marker. 1: pCAMBIA0380-*ApSDR53C2* (10799 bp); 2: pCAMBIA0380 empty plasmid vector (6813 bp); 3: pCAMBIA0380-*ApSDR53C2* double digested product by ApaI and Hind III restriction enzymes (6773 bp and 4026 bp); 4: *ApSDR53C2*-5-hph-*ApSDR53C2*-3 DNA fragment (4026 bp). (B) Knockdown vectors pCAMBIA0380-*ApSDR548U2* were enzymatically verified. Note: M: DL15000 DNA marker. 1: pCAMBIA0380- *ApSDR548U2* (10799 bp); 2: pCAMBIA0380 empty plasmid vector (6813 bp); 3: pCAMBIA0380- *ApSDR548U2* double digested product by ApaI and Hind III restriction enzymes (6773 bp and 4026 bp); 4: *ApSDR548U2* -5-hph- *ApSDR548U2* -3 DNA fragment (4026 bp). (C-E) The maps of the empty plasmid vector, pCAMBIA0380-*ApSDR53C2* vector and pCAMBIA0380-*ApSDR548U2* vector.


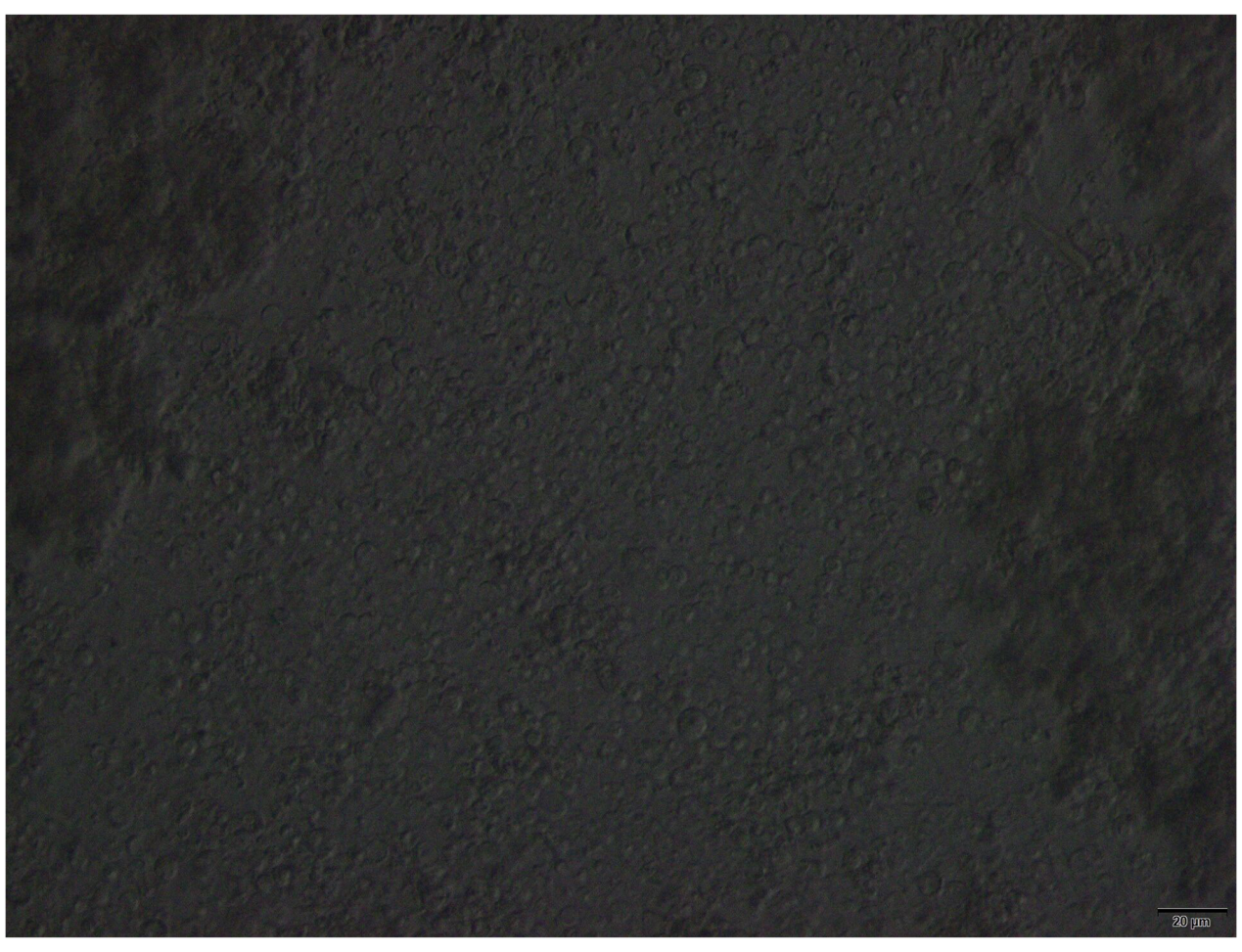


**Figure S6.** Morphology of the protoplasts of *A. phaeospermum* under 10×40x light microscope.


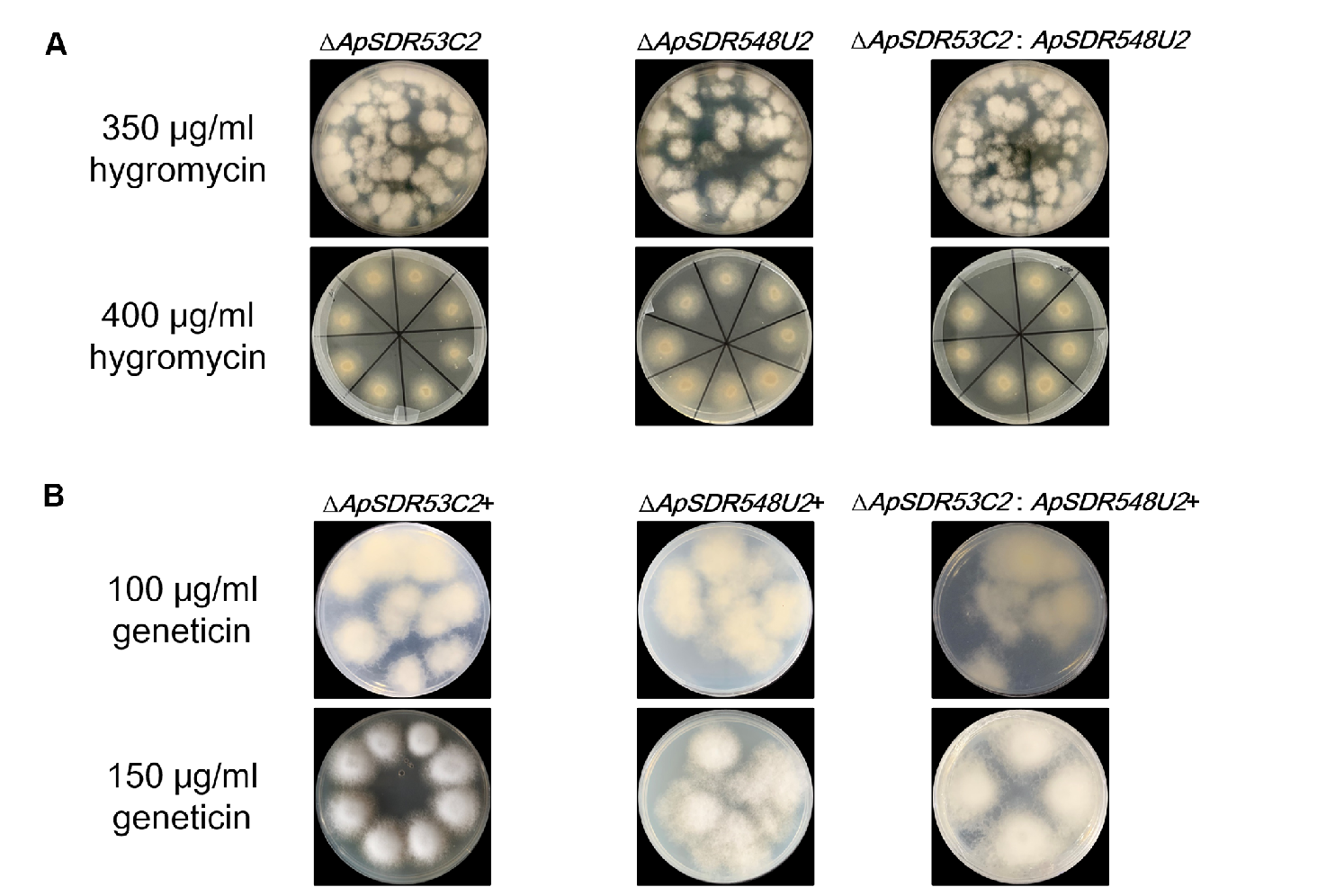


**Figure S7.** Colony diagrams of transformants. (A) Colonies grown under 350 µg/mL of hygromycin were inoculated onto PDA supplemented with 400 µg/mL of hygromycin, and the colonies grown under 400 µg/mL of hygromycin were positive knock-out transformants. (B) Colonies grown under 100 µg/mL of geneticin were inoculated onto PDA supplemented with 150 µg/mL of geneticin, and the colonies grown under 150 µg/mL of geneticin were positive complemented transformants.


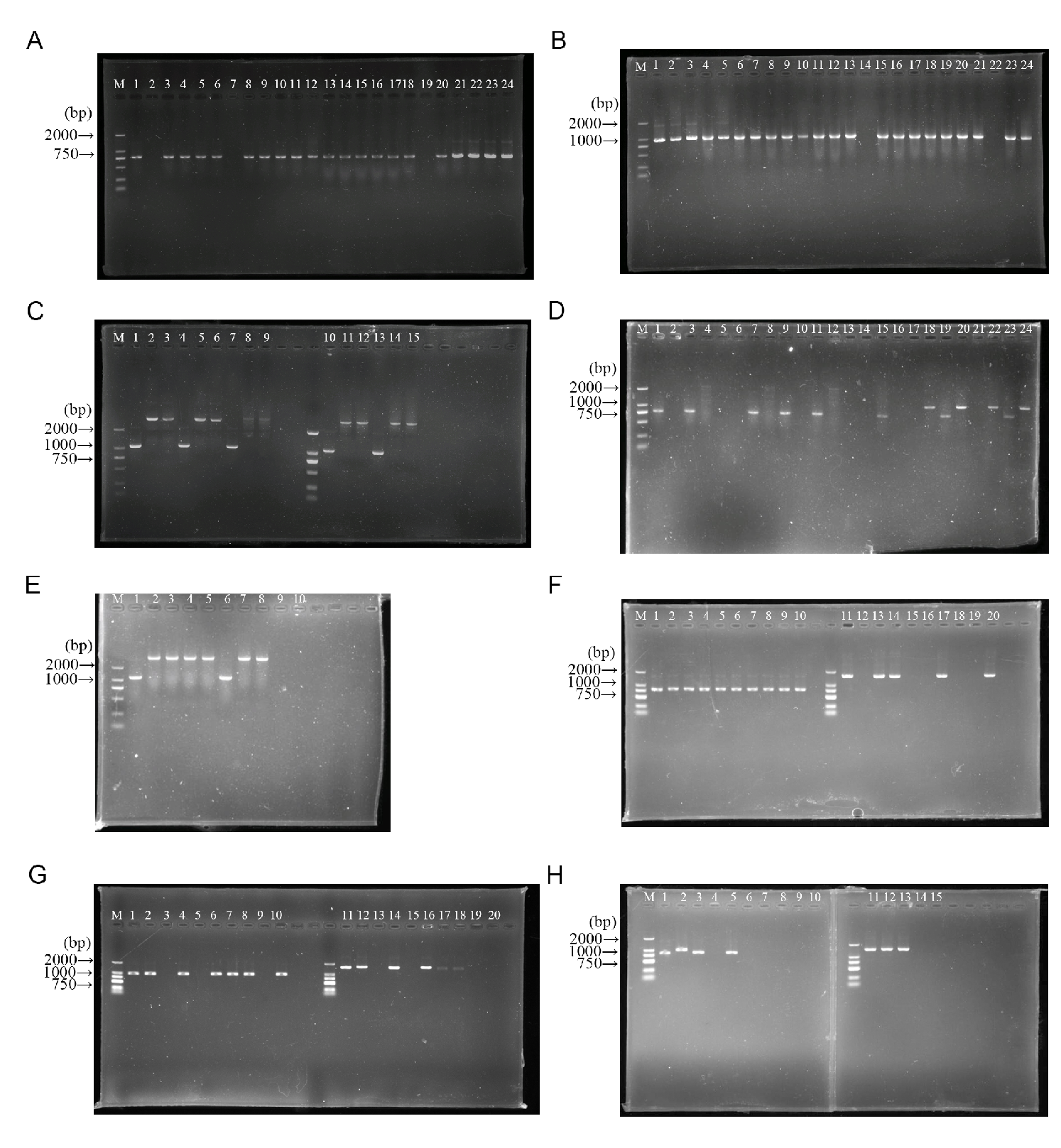


**Figure S8.** Results of PCR assay of positive transformants. (A) Electropherogram of the first round of PCR assay for *ApSDR53C2* knockout transformants. Note: M: DL2000 DNA marker. 1-24: *ApSDR53C2* gene (789 bp) of *ApSDR53C2* knockout transformant strains. (B) Electropherogram of the first round of PCR assay for *ApSDR548U2* knockout transformants. Note: M: DL2000 DNA marker. 1-24: *ApSDR548U2* gene (891 bp) of *ApSDR548U2* knockout transformant strain. (C) Electropherogram of the second round of PCR assay for *ApSDR53C2* and *ApSDR548U2* knockout transformants. Note: M: DL2000 DNA marker. 1-9: hph gene (1026 bp), *ApSDR53C2*-5-hph DNA fragment (2526 bp), and *ApSDR53C2*-3-hph DNA fragment (2526 bp) of *ApSDR53C2* knockout transformant strains. 10-15: hph gene (1026 bp), *ApSDR548U2*-5-hph DNA fragment (2526 bp), and *ApSDR548U2*-3-hph DNA fragment (2526 bp) of *ApSDR548U2* knockout transformant strains. (D) Electropherogram of the first round of PCR assay for *ApSDR53C2*:*ApSDR548U2* co-knockout transformants. Note: M: DL2000 DNA marker. Odd-numbered lanes: *ApSDR53C2* gene (789 bp) of *ApSDR53C2*:*ApSDR548U2* co*-*knockout transformant strains. Even numbered lanes: *ApSDR548U2* gene (891 bp) of *ApSDR53C2*:*ApSDR548U2* co*-*knockout transformant strains. (E) Electropherogram of the second round of PCR assay for *ApSDR53C2*:*ApSDR548U2* co-knockout transformants. Note: M: DL2000 DNA marker. 1 and 6: hph gene (1026 bp) of *ApSDR53C2*:*ApSDR548U2* co*-*knockout transformant strains. 2-5 and 7-10: *ApSDR53C2*-5-hph DNA fragment (2526 bp), *ApSDR53C2*-3-hph DNA fragment (2526 bp), *ApSDR548U2*-5-hph DNA fragment (2526 bp), and *ApSDR548U2*-3-hph DNA fragment (2526 bp) of *ApSDR53C2*:*ApSDR548U2* co*-*knockout transformant strains. (F) Electropherogram of the PCR assay for *ApSDR53C2* complemented transformants. Note: M: DL2000 DNA marker. 1-10: *ApSDR53C2* gene (789 bp). 11-20: kanMx gene (1357 bp). (G) Electropherogram of the PCR assay for *ApSDR548U2* complemented transformants. Note: M: DL2000 DNA marker. 1-10: *ApSDR548U2* gene (891 bp). 11-20: kanMx gene (1357 bp). (H) Electropherogram of the PCR assay for *ApSDR53C2*:*ApSDR548U2* co-complemented transformants. Note: M: DL2000 DNA marker. Odd-numbered lanes of 1-10: *ApSDR53C2* gene (789 bp). Even numbered lanes of 1-10: *ApSDR548U2* gene (891 bp). 11-15: kanMx gene (1357 bp).

**Table S1.** Primers of six candidate *ApSDRs* and internal reference gene (Tubulin) for qPCR.

| **Primer Name** | **Primer Sequence 5’-3’** |
| --- | --- |
| *ApSDR53C2* -F | CCCGAGCCTGAGGAACAAAA |
| *ApSDR53C2*-R | AGAAATTGTGGCTGCGAGGA |
| *ApSDR548U2*-F | CGCGTATTGTGGTCGTGTCT |
| *ApSDR548U2*-R | GGTGGCGCTTGTTGTCTTG |
| *ApSDR58C1*-F | TTCTGTGCAGTGGTCTGGTC |
| *ApSDR58C1*-R | ATGTAAGGTTCTCGACCCGC |
| *ApSDR118C3*-F | GGGAGGTGGACAGGAAGTTG |
| *ApSDR118C3*-R | CACTGTCGTCTTCAAGGGCT |
| *ApSDR472C1*-F | CTTCCGTCCCATCGGCAACA |
| *ApSDR472C1*-R | TCGCTTTGCCGATACCCTG |
| *ApSDR86C6*-F | ATTTGCTCGGTCTGGCAGTT |
| *ApSDR86C6*-R | CCTGGGCTTGAGGGTTGATT |
| Tubulin-F | CTACAACGGTACCTCGGAGC |
| Tubulin-R | ACCGGATTGACCGAAAACGA |

**Table S2.** Up and downstream homologous arm primers of hph and target gene.

| **Primer Name** | **Primer Sequence 5’-3’** |
| --- | --- |
| *ApSDR53C2*-5-F | TAGGCCACCATGTTGGGCCCCTGGCGTTGAACTGGCC |
| *ApSDR53C2-*5-R | AGTTCAGGCTTTTTCATATCTGTTATTAGCCAGCAGAAATCTCGG |
| *ApSDR53C2-*3-F | CGAGGGCAAAGGAATAGAGTTCGACCGGGGGGGCTCAA |
| *ApSDR53C2-*3-R | GTGGACTCCTCTTAAAGCTTGTTTACACCATAATGGTATACTGCCTG |
| *ApSDR548U2-*5-F | AGTTCAGGCTTTTTCATATCGATTAATGGTGGATCTACTGAAAG |
| *ApSDR548U2*-5-R | GCGCCGAATTCCCGGGGATCCGGCATTTGGAGATTGAGAAAC |
| *ApSDR548U2-*3-F | TGGCTGCAGGTCGACGGATCCCAGAGCCTCCCCGAAAACG |
| *ApSDR548U2-*3-R | CGAGGGCAAAGGAATAGAGTGCCTGGGGACAAAAAATAAGTG |
| *Hph*-F | GATATGAAAAAGCCTGAACT |
| *Hph*-R | ACTCTATTCCTTTGCCCTCG |
| KanMX-F | CAGCTGAAGCTTCGTACGC |
| KanMX-R | GCATAGGCCACTAGTGGATCTG |

**Table S3.** Disease grading standard.

| **Grade** | **Symptomatic** |
| --- | --- |
| 0 | no wilt |
| 1 | less than 25% of branches withered |
| 2 | 25%-50% (including 25% and 50%) of branches withered |
| 3 | 50%-75% of branches dead (including 75%) |
| 4 | more than 75% of branches withered |

**Table S4.** The internal primers of each melanin gene and reference gene (Tubulin) for qPCR.

| **Primer Name** | **Primer Sequence 5’-3’** |
| --- | --- |
| *ApCmr1*-F | \| CCACCACCTTGGATACTCCG \| \| --- \| |
| *ApCmr1*-R | GTGGTTGATGTAAGCCCGTCA |
| *ApLac1*-F | \| GAGGCGGCCATGAAGTTCTA \| \| --- \| |
| *ApLac1*-R | ACCGTTAGGACTGATCGCTT |
| THN-reductase-F | \| CTGTGGTATCGTGGACGAGG \| \| --- \| |
| THN-reductase-R | CTTGTTGGTGAGGCCAAACG |
| *ApPKS15*-F | AAGTCTCGACCTCTCGCTTG |
| *ApPKS15*-R | \| GGTATCGAGAAGTGGCGAGG \| \| --- \| |
| *ApPKS21*-F | \| GGATCAAGACGCAGAGCAGA \| \| --- \| |
| *ApPKS21*-R | GCGGTCAGACTTCCAGTGT |
| T4HN-reductase-F | GACAGGTAGAGCGCCTTCTC |
| T4HN-reductase-R | \| GGCTGACCTGTATGCGTAGG \| \| --- \| |
| Tubulin-F | CTACAACGGTACCTCGGAGC |
| Tubulin-R | ACCGGATTGACCGAAAACGA |
